# RAD51-targeting small molecule degrader sensitizes *BRCA*-proficient prostate cancer cells to PARP inhibitors *via* synthetic lethality

**DOI:** 10.1016/j.apsb.2026.03.029

**Published:** 2026-03-19

**Authors:** Yanlin Jian, Yibo Gao, Tianyang Zhou, Shan Xu, Bin Wang, Yizeng Fan, Jian Ma, Yang Gao, Jing Liu, Bohan Ma, Lei Li

**Affiliations:** Department of Urology, The First Affiliated Hospital of Xi'an Jiaotong University, Xi'an 710061, China

**Keywords:** RAD51, Degrader, BRCA2, Homologous recombination, PARP inhibitors, Synthetic lethality, Resistance, Prostate cancer

## Abstract

Poly (ADP-ribose) polymerase (PARP) inhibitors though effective in patients with homologous recombination (HR)-deficient tumors, a large population of patients remain unresponsive, primarily due to either the absence of HR-related mutation or the restoration of HR functionality. RAD51, a critical protein in HR repair signaling that ensures precise DNA lesion repair, represents a promising therapeutic target. Inspired by the clinical success of PARP inhibitors in treating *BRCA1/2*-mutant cancers and leveraging the potential of proteolysis-targeting chimeras (PROTAC) technology—a method that exploits the cell's protein degradation machinery to eliminate disease-associated proteins, we generated a small-molecule PROTAC G73. This compound degrades RAD51 in a concentration- and time-dependent manner, effectively mimicking the HR-deficient phenotype by impairing DNA double-strand break (DSB) repair. Furthermore, G73-mediated RAD51 degradation synergizes with the PARP inhibitor olaparib, inducing synthetic lethality and re-sensitizing olaparib-resistant cancers to PARP inhibition. This fully small-molecule-based strategy presents a compelling strategy to overcome resistance to PARP inhibitors, expanding their therapeutic potential beyond patients with HR-deficient tumors.

## Graphical abstract

RAD51 PROTAC G73 blocks proliferation and synergizes PARP inhibitors in HR-proficient prostate cancer, extending the clinical application of PARP inhibitors for HR-proficient mCRPC treatment.

**Figure:**
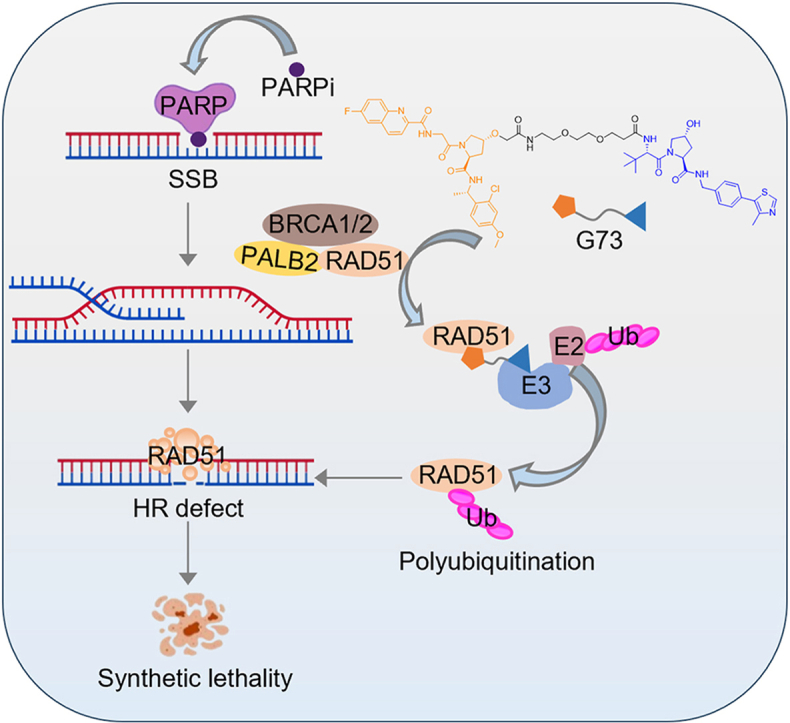

## 1 Introduction

Despite advances in early detection and initial treatment modalities, metastatic castration-resistant prostate cancer (mCRPC) remains a formidable clinical challenge due to its intrinsic heterogeneity, rapid progression, and the eventual failure of standard androgen deprivation therapies^1^, ^2^, ^3^, ^4^. Recent research has increasingly focused on elucidating the molecular and genetic mechanisms underpinning treatment resistance in mCRPC, leading to the exploration of poly (ADP-ribose) polymerase (PARP) inhibitors as potential therapeutic options for patients with specific genetic alterations, particularly those with defects in DNA repair pathways^5^, ^6^, ^7^, ^8^. In clinical trials evaluating PARP inhibitors for advanced prostate cancer, patients harboring *BRCA2* mutations have demonstrated superior drug responses compared to those with *BRCA1* mutations, suggesting that the BRCA2-mediated pathway plays a more prominent role in the DNA repair process within prostate cancer^9^^,^^10^. Mechanistically, BRCA2 interacts with RAD51 and facilitates its loading onto single-stranded DNA at double-strand break (DSB) sites, which is a critical step in homologous recombination (HR) repair^11^^,^^12^. The loss of BRCA2 function consequently leads to HR defects, rendering cancer cells more susceptible to the synthetic lethality induced by PARP inhibition. This paradigm not only underscores the significance of the BRCA2/RAD51 axis in maintaining genomic integrity but also highlights the potential of exploiting HR deficiencies as a therapeutic strategy in prostate cancer and other malignancies. However, mutations in *BRCA2* are observed in only a small percentage of men with prostate cancer, with reported prevalence around 13%^13^. Expanding the scope of PARP inhibitors use beyond this limited population is an emerging direction in prostate cancer treatment.

The BRCA2/RAD51 complex plays a pivotal role in mediating HR repair^11^^,^^14^, ^15^, ^16^. As an ATPase, RAD51 forms a nucleoprotein filament on single-stranded DNA, which is essential for the search and invasion of homologous sequences—a key step that allows the cell to accurately repair DSBs and other forms of DNA damage^17^. Additionally, RAD51 is involved in stabilizing replication forks during DNA synthesis, thereby preventing fork collapse—a common precursor to genomic instability^18^^,^^19^. This role is particularly crucial during periods of replicative stress, where timely and efficient repair is needed to ensure accurate DNA replication and cell survival. Clinically, the importance of RAD51 extends to its potential as a biomarker and therapeutic target^20^^,^^21^. Tumors that exhibit high levels of RAD51 often display increased repair proficiency, contributing to their survival and resistance to therapies that induce DNA damage. Conversely, targeting RAD51 could emerge as a promising strategy to sensitize cancer cells to PARP inhibitors. This approach is particularly relevant in the context of synthetic lethality^22^, ^23^, ^24^, ^25^, ^26^, where the inhibition of RAD51-mediated repair in cells already deficient in other repair mechanisms can lead to selective cancer cell death. To this end, structure-based strategy has yielded several peptidomimetics^27^^,^^28^ and non-peptide inhibitors^24^, ^25^, ^26^^,^^29^, ^30^, ^31^, ^32^, ^33^, ^34^, ^35^, ^36^, ^37^ (Supporting Information Fig. S1) with sub-micromolar to micromolar range inhibitory activity, earmarking RAD51 as a promising candidate cancer drug target.

Given the central role of RAD51 in homologous recombination and its contribution to DNA repair proficiency in cancer cells, this study aims to discover more potent RAD51-targeting agents by developing a proteolysis-targeting chimera (PROTAC) against RAD51. By harnessing the targeted protein degradation capabilities of PROTAC technology, the RAD51 targeting PROTAC drug selectively eliminates RAD51 from cancer cells, thereby disrupting a critical repair mechanism and sensitizing the cells to the DNA-damaging effects of PARP inhibitors. As a result, the combination of RAD51 PROTAC with PARP inhibitors could overcome inherent resistance mechanisms and enhance therapeutic efficacy, potentially leading to improved clinical outcomes. In summary, the development of RAD51 PROTAC drug offers a groundbreaking therapeutic avenue by effectively sensitizing cancer cells to PARP inhibition. This strategy underscores the importance of targeting critical DNA repair proteins and opens new horizons for the treatment of aggressive malignancies, such as mCRPC, where treatment options remain limited.

## 2 Materials and methods

### 2.1 Cell lines, culture conditions, antibodies and plasmids

The human HEK293T, PC-3 and C4-2 cell lines were grown in RPMI-1640 or DMEM medium supplemented with 10% FBS (Fetal Bovine Serum) and 1% Penicillin–Streptomycin at 37 °C with 5% CO_2_. The anti-*γ*-H2AX (9718 T) antibody was purchased from Cell Signaling Technology (Danvers, MA, USA). The anti-RAD51 (ab133534) antibody and anti-Ki67 (ab15580) antibody were purchased from Abcam (Cambridge, UK). The anti-VINCULIN antibody (V-4505), anti-HA agarose beads (A-2095), secondary anti-mouse antibody, and secondary anti-rabbit antibody were purchased from Sigma-Aldrich (St. Louis, MO, USA). The plasmids Flag-RAD51 (P10470) and HA-VHL (P60536) were purchased from MiaoLingBio (Wuhan, China).

### 2.2 CCK-8 cell viability assay

Cell viability was assessed using the Cell Counting Kit-8 (CCK-8). Cells were seeded at a density of 3 × 10^3^ cells per well on a 96-well plate, with three independent samples for each concentration. After 48 h of treatment with indicated compounds, cell viability was measured. Following a 2 h incubation with CCK-8 reagent, the absorbance value at 450 nm was measured with a microplate reader. The CCK-8 reagent kit (CK04) was purchased from Dojindo Laboratories (Shanghai, China).

### 2.3 Immunoblotting

Cells were lysed with RIPA buffer (100 mmol/L Tris–HCl, pH 7.4, 150 mmol/L NaCl, 1 mmol/L EDTA, 1% Triton X-100, 1% sodium deoxycholate, 0.1% SDS) containing protease and phosphatase inhibitors. Denatured proteins were separated by SDS-PAGE gels and transferred to a nitrocellulose membrane. Membranes were blocked with 5% milk at room temperature for 1 h, followed by overnight incubation with primary antibodies. After incubation with secondary antibodies at room temperature for 1 h, protein bands were visualized using the ECL detection system (ThermoFisher Scientific, Rochester, USA) and exposed to X-ray film.

### 2.4 Co-immunoprecipitation

Cells transfected with plasmids were treated with indicated compound for 24 h and lysed with IP buffer (50 mmol/L Tris-HCl, 150 mmol/L NaCl, 1 mmol/L EDTA, 1% Triton X-100) containing protease and phosphatase inhibitors. Proteins were incubated with HA agarose beads at 4 °C for 4 h. The cell lysates were extensively washed with IP buffer, then boiled at 100 °C for 5 min to elute the protein from the beads. Proteins were separated by SDS-PAGE and analyzed by western blotting.

### 2.5 Immunofluorescence (IF)

PC-3 cells were seeded on 13-mm glass coverslips. After 24 h of treatment, cells were washed once with cold PBS and fixed with 4% formaldehyde in PBS at room temperature for 15 min. 0.2% Triton X-100 in PBS was used to permeabilize the cells for 15 min. After three washes, the cells were incubated with primary antibodies in 5% BSA and 5% glycerol in PBS. After overnight incubation at 4 °C, the samples were washed three times with PBS and incubated with fluorescently coupled antibodies for 1 h at room temperature. After three washes, the coverslips were stained with 10 mg/mL 4ʹ,6-diamidino-2-phenylindole for 5 min, mounted on slides and visualized using a fluorescence microscope.

### 2.6 Colony formation assay

Cells were seeded at a density of 1 × 10^3^ cells per well on 6-well plates and allowed to grow in a medium containing indicated compound. After 7 days, the colonies were fixed with 4% paraformaldehyde for 15 min and then stained with 0.5% crystal violet for 30 min. Colonies containing more than 50 cells were counted after being gently washed with running water.

### 2.7 Antitumor activity in PC-3 xenograft model

Treatment was initiated when palpable tumors (approximately 50 mm^3^) formed in a xenograft model of PC-3 cells, 2 weeks after inoculation. Mice were randomly divided into five treatment groups (5 per group) and administered with indicated treatments every other day for 3 weeks, starting on Day 1. Tumor length and width were measured with a caliper, and tumor volume was calculated using Eq. (1):

(1) Tumor volume (*V*) = Length × Width^2^/2

The animals were euthanized at the end of the experiment. Immunohistochemical staining of RAD51, Ki67 and *γ*-H2AX were conducted in tumor tissue sections. Immunohistochemistry staining images were analyzed and scored using the ImageJ profiler, following published protocols.

### 2.8 Ethical approval

All experimental procedures involving animals were conducted by institutional guidelines and were approved by the Laboratory Animal Center and Biomedical Ethics Committee of Xi'an Jiaotong University (Approval No: XJTUAE2023-1865).

### 2.9 Quantification and statistical analysis

Graphs were generated using GraphPad Prism 8 (GraphPad, Inc.) or Microsoft Office Excel 2016. Data are represented as mean ± standard deviation (SD). Statistical comparisons between groups were performed using *t*-tests or one-way ANOVA by GraphPad Prism 8. A *P*-value of <0.05 was considered statistically significant. The sample size was not predetermined by using statistical methods.

## 3 Results

### 3.1 The development of effective PROTACs to facilitate RAD51 protein degradation

**Figure 1:**
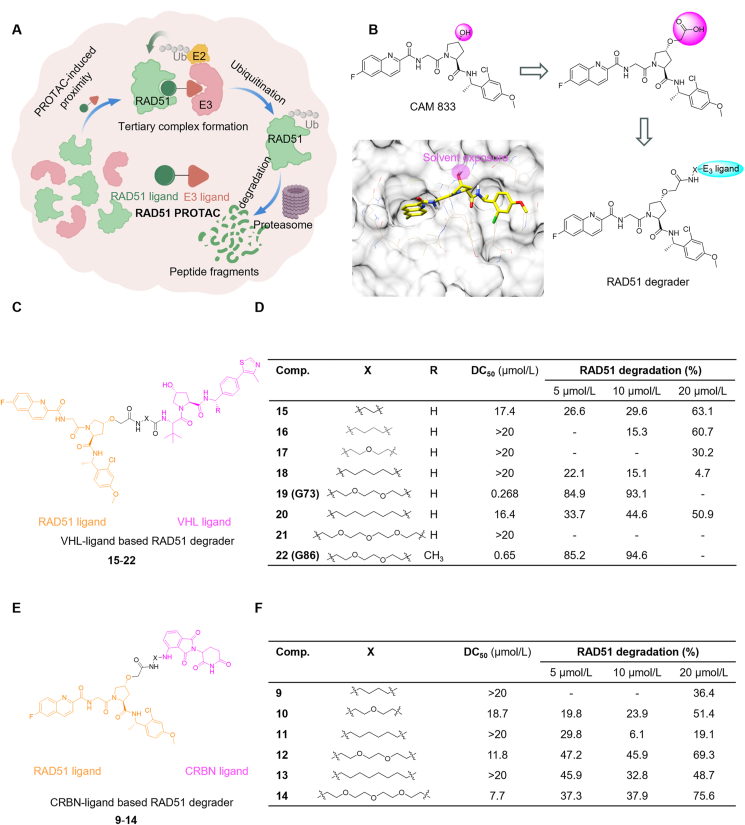
Design and development of RAD51 degraders. (A) Schematic diagram of RAD51 PROTAC consisting of RAD51 and E3 warheads, bringing RAD51 and E3 into proximity to facilitate RAD51 degradation. (B) Design of RAD51 degraders based on the cocrystal structure of CAM833 in complex with RAD51 (PDB: 6TW9). (C) The structures of envisioned VHL-based RAD51 degraders. (D) The effects of synthesized compounds on the RAD51 protein level in PC-3 cells. –, no degradation. (E) The structures of envisioned CRBN-based RAD51 degraders. (F) The effects of synthesized compounds on the RAD51 protein level in PC-3 cells. –, no degradation.

RAD51 contributes to tumor initiation and development across multiple cancer types, with elevated RAD51 protein expression linked to poor clinical outcomes. Notably, RAD51 expression is higher in prostate cancer cell lines than in nonneoplastic cells (Supporting Information Fig. S2A), highlighting its potential as a promising therapeutic target for prostate cancer. To discover molecules that could effectively interfere with RAD51 protein, we set out to identify heterobifunctional RAD51 degraders. Through the two warheads to recruit RAD51 and E3 individually, the envisioned degrader induces proximity to facilitate the degradation of RAD51 protein (Fig. 1A). We utilized the small molecule CAM833 as the warhead, which exhibits sub-micromolar affinity^37^. The cocrystal structure of CAM833 in complex with RAD51 revealed that the hydroxy group on the pyrrolidine protrudes outside the binding pocket and is solvent-exposed (Fig. 1B), making it an ideal site for linker attachment. The hydroxy moiety was alkylated with *tert*-butyl bromoacetate to convert it into a more synthetically amenable acetic group. By conjugating this modified molecule with ligands targeting either the E3 ligase von Hippel-Lindau (VHL) or cereblon (CRBN) *via* various linkers, two series of molecules were created: VHL-ligand-based RAD51 degraders and CRBN-ligand-based RAD51 degraders (Fig. 1C and E, Supporting Information Schemes S1 and S2). These compounds were evaluated for their ability to induce RAD51 protein degradation in PC-3 cancer cells. Among the envisioned compounds, **19** (G73) and **22** (G86), bearing the VHL ligand, demonstrated significant efficacy, reducing RAD51 protein levels by over 80% at 5 μmol/L after 24 h of treatment in PC-3 cell lines (Fig. 1D and F, Fig. S2B and S2C).

**Figure 2:**
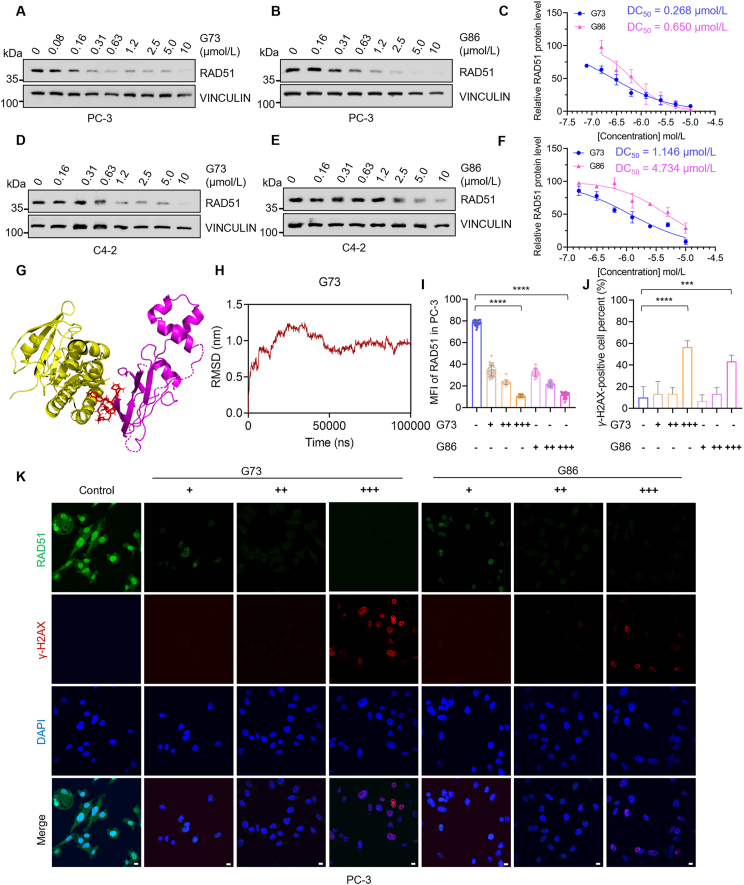
G73 and G86 effectively induce the degradation of RAD51 *in vitro.* (A, B) Immunoblotting (IB) analysis of RAD51 protein in PC-3 cells after 24 h treatment of G73 (A) and G86 (B) at a concentration ranging from 0 to 10 μmol/L. (C) Degradation curve of G73 and G86 in PC-3 cells. (D, E) IB analysis of RAD51 protein in C4-2 cells after 24 h treatment of G73 (D) and G86 (E) at a concentration ranging from 0 to 10 μmol/L. (F) Degradation curve of G73 and G86 in C4-2 cells. (G) The predicted binding mode of the RAD51: G73: VHL ternary complex (yellow: RAD51, magenta: VHL). (H) The RMSD curve of the ternary complex induced by G73. (I, J) Quantification of RAD51 mean fluorescence intensity (MFI) per field (I) and the percentage of *γ*-H2AX-positive cells (≥5 foci, J) after 24 h treatment of G73 and G86. +, ++ and +++ represent 1, 2 and 5 μmol/L G73/G86 respectively. The data are presented as the mean ± SD values (*n* = 30). Ordinary one-way ANOVA. ∗∗∗*P* < 0.001, ∗∗∗∗*P* < 0.0001 *vs*. indicated (K) Representative immunofluorescence (IF) staining of RAD51 and *γ*-H2AX in PC-3 cells after 24 h treatment of G73 and G86 at indicated concentration. Scale bar = 10 μm. +, ++ and +++ represent 1, 2 and 5 μmol/L G73/G86 respectively.

Subsequently, the efficacy of G73/G86 in reducing RAD51 protein levels was further investigated across various cell lines. Both G73 and G86 significantly reduced RAD51 protein in a dose-dependent manner in PC-3 and C4-2 cells. The half-maximal degradation concentration (DC_50_) was determined to be 0.268 μmol/L/1.146 μmol/L (PC-3/C4-2) for G73 and 0.650 μmol/L/4.734 μmol/L for G86 (Fig. 2A‒F). To elucidate the binding mechanism, we conducted comprehensive molecular docking and molecular dynamics (MD) simulations for the proposed compounds. Our modeling approach involved first anchoring the docking structure of one protein to the bifunctional molecule, followed by systematic docking of the second protein to simulate trimer formation. Analysis of the resulting RMSD trajectory confirmed system equilibrium, while detailed examination of the ternary complex structure revealed specific molecular interactions. The docking results demonstrated that compound G73 exhibits superior binding characteristics compared to less active compound **20**, effectively engaging both RAD51 and VHL simultaneously (Fig. 2G, Fig. S2D and S2E). These findings confirm G73's ability to successfully recruit VHL to RAD51, facilitating formation of a stable RAD51: G73: VHL ternary complex. The predicted stability of this complex was further validated through MD simulations (Fig. 2H), supporting the structural viability of the proposed interaction mechanism. To verify that the degradation of RAD51 protein was dependent on the ternary complex formation induced by the PROTAC rather than the warheads, the RAD51 inhibitor CAM833 was assayed for its ability to reduce RAD51 protein levels. The results showed that CAM833 had minimal impact on RAD51 protein levels (Fig. S2F). Moreover, immunofluorescence imaging further confirmed RAD51 protein degradation induced by G73 and G86 (Fig. 2I and K). Additionally, the treatment with G73 and G86 resulted in the formation of *γ*-H2AX foci, indicating that RAD51 protein degradation mediated by these compounds led to persistent HR inhibition and increased DNA damage^35^ (Fig. 2J). Taken together, these findings demonstrate that G73 and G86 are promising RAD51 degraders with the potential to induce DNA damage.

**Figure 3:**
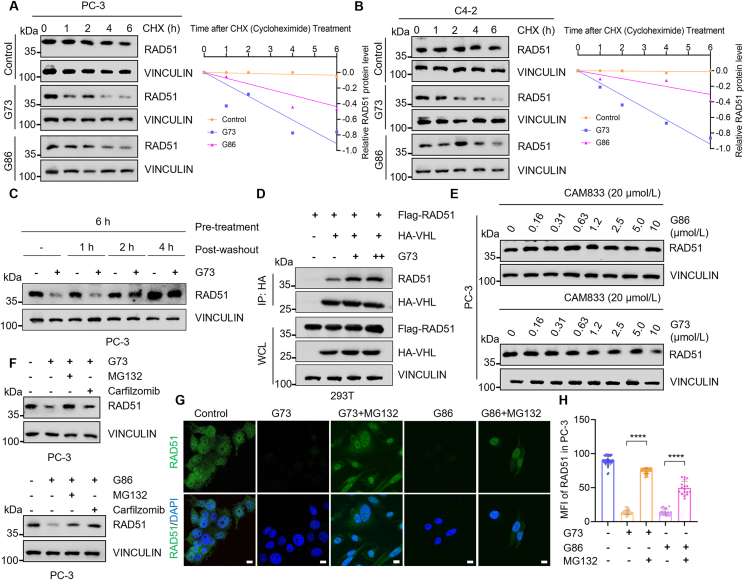
RAD51 PROTAC induces degradation in a time- and VHL-dependent manner. (A, B) IB analysis of RAD51 in PC-3 (A) and C4-2 (B) cells treated with CHX for the indicated time, with or without G73/G86 treatment (5 μmol/L). The signal intensities of RAD51 normalized to that of VINCULIN were quantified. (C) The G73-induced degradation of RAD51 is reversible. PC-3 cells were pretreated with G73 (5 μmol/L) or vehicle for 6 h, washed with PBS, and cultured in fresh media (without G73) for the indicated hours. (D) Co-immunoprecipitation (IP) analysis of HA-VHL and Flag-RAD51 in 293 T cells transfected with indicated plasmids, with or without G73 (+: 2.5 μmol/L, ++: 5 μmol/L) treatment. (E) IB analysis of RAD51 protein in PC-3 cells after pretreatment with RAD51 inhibitor CAM833 (20 μmol/L), followed by 24 h treatment of G73/G86. (F) IB analysis of RAD51 in PC-3 cells with or without pretreatment with MG132 (10 μmol/L)/Carfilzomib (10 nmol/L), followed by G73/G86 treatment (5 μmol/L). (G, H) Representative IF staining of RAD51 and corresponding MFI quantification in PC-3 cells with or without MG132 (10 μmol/L) pretreatment before G73/G86 (5 μmol/L) treatment. Scale bar = 10 μm. The data are presented as the mean ± SD values (*n* = 17). Ordinary one-way ANOVA. ∗∗∗∗*P* < 0.0001 *vs*. indicated.

Next, the kinetics of G73/G86-induced RAD51 degradation was investigated. Cotreatment with protein synthesis inhibitor cycloheximide (CHX) revealed that G73 and G86 specifically accelerated protein degradation without affecting RAD51 protein synthesis in PC-3 and C4-2 cells (Fig. 3A and B). We also performed a washout experiment for G73. As illustrated in Fig. 3C, G73-induced RAD51 degradation persisted for 1 h after the removal of G73 from the culture medium, with RAD51 protein levels returning to baseline within 4 h. This indicates that the reduction in RAD51 protein levels induced by G73 is reversible. To investigate the cellular mode of action of G73, its capacity to facilitate interactions between RAD51 and VHL in the 293 T cell line was assessed. The 293 T cells transfected with Flag-RAD51 or HA-VHL plasmids were treated with 2.5/5 μmol/L G73 for 24 h, followed by lysis for immunoprecipitation analysis. As shown in Fig. 3D, RAD51 displayed a weak basal affinity for VHL under physiological conditions, which was significantly enhanced upon G73 treatment, leading to RAD51 protein degradation. This effect was diminished when cells were pretreated with the RAD51 inhibitor CAM833 (Fig. 3E). Correspondingly, the G73-induced degradation of RAD51 protein was abolished when cells were co-treated with the proteasome inhibitors MG132 or Carfilzomib (Fig. 3F‒H). These findings collectively suggest that G73 promotes RAD51 degradation by recruiting E3 ligase VHL to RAD51, enabling subsequent ubiquitination.

### 3.2 G73 enhances DNA damage and exhibits synergistic effects with the PARP inhibitor olaparib

**Figure 4:**
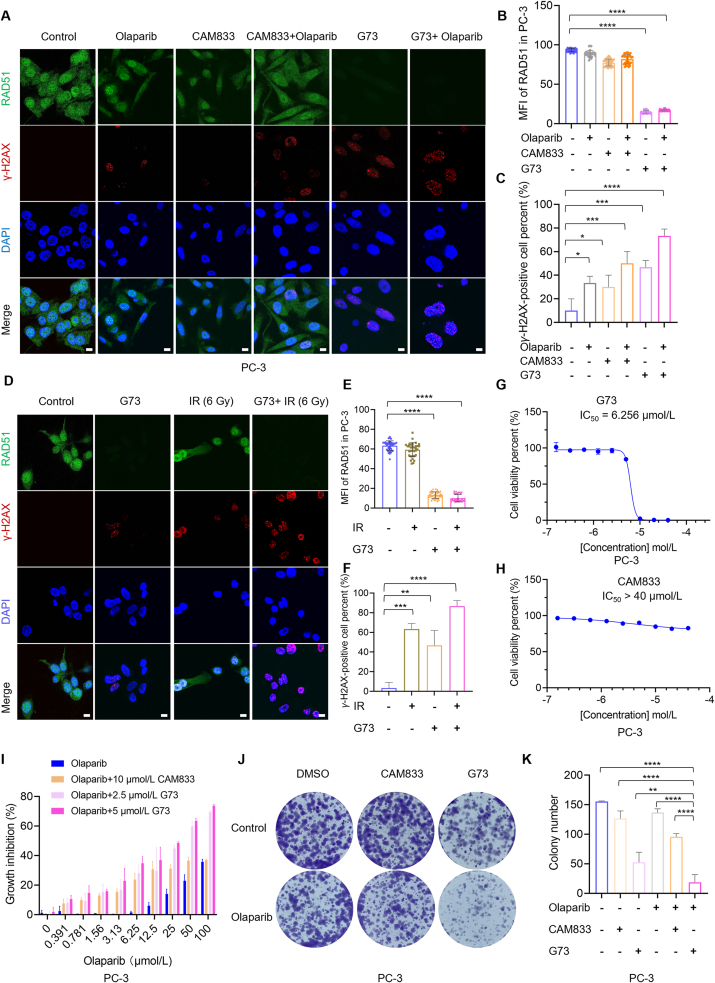
G73 synergizes with olaparib to attenuate HR activity. (A) Representative IF staining of RAD51 and *γ*-H2AX in PC-3 cells after indicated drug treatment. Scale bar = 10 μm. CAM833: 10 μmol/L; olaparib: 10 μmol/L; G73: 5 μmol/L. (B, C) RAD51 MFI per field (B) and *γ*-H2AX foci positive cell percent (≥5 foci, C) in PC-3 cells were quantified. The data are presented as the mean ± SD values (*n* = 30). Ordinary one-way ANOVA; ∗*P* < 0.05, ∗∗∗*P* < 0.001; ∗∗∗∗*P* < 0.0001 *vs*. indicated. (D) Representative IF staining of RAD51 in PC-3 cells with or without G73 treatment (5 μmol/L), harvested after IR (6 Gy) for 1 h, Scale bar = 10 μm. (E, F) RAD51 MFI per field (E) and *γ*-H2AX positive cell percent (≥5 foci, F) in PC-3 cells were quantified. The data are presented as the mean ± SD values (*n* = 30). Ordinary one-way ANOVA; ∗∗*P* < 0.01, ∗∗∗*P* < 0.001, ∗∗∗∗*P* < 0.0001 *vs*. indicated. (G‒I) Cell viability of PC-3 cells after 48 h treatment with varying concentrations of indicated drug. (J, K) Colony formation in PC-3 cell lines treated with CAM833 (10 μmol/L), G73 (5 μmol/L) or olaparib (2.5 μmol/L), respectively or simultaneously. The number of colonies was counted. Representative colonies are shown in (J), with quantification data shown in (K). The data are presented as the mean ± SD values (*n* = 3). Ordinary one-way ANOVA. ∗∗*P* < 0.01; ∗∗∗∗*P* < 0.0001 *vs*. indicated.

Studies have shown that RAD51 is recruited and assembles into microscopic foci upon DNA damage^38^. By degrading RAD51, G73 is anticipated to disrupt its assembly on DNA substrates, thereby increasing DNA damage. To evaluate the impact of G73 on HR activity, the extent of DNA damage in PC-3 cells treated with CAM833 or G73, either alone or in combination with the PARP inhibitor olaparib, was assessed by monitoring nuclear *γ*-H2AX foci through immunofluorescence. The results, depicted in Fig. 4A‒C, demonstrated a marked increase in *γ*-H2AX labeling in the nuclei of PC-3 cells exposed to the combined treatments (CAM833+olaparib or G73+olaparib) compared to those treated with CAM833, G73, or olaparib individually. Furthermore, this effect was more pronounced following exposure to 6 Gy ionizing radiation (IR, Fig. 4D‒F). These results collectively support the proposed mechanism for G73.

Subsequent experiments were conducted to determine whether coadministration of G73 and olaparib induces synthetic lethality. Cell viability was measured 48 h after treatment of PC-3 cells with CAM833 or G73, either alone or in combination with olaparib. The results indicated that G73 suppresses PC-3 cell growth in a concentration-dependent manner, outperforming CAM833 (Fig. 4G and H). This antiproliferation effect of G73 was also validated in other prostate cancer cell lines, C4-2 and 22Rv1 (Supporting Information Fig. S3A‒S3C). Moreover, our results showed that G86 was less effective than G73 on inhibiting cancer cell proliferation (Fig. S3D and S3E). Given that cells deficient in RAD51-mediated homology dependent repair (HDR) due to inactivation of tumor suppressor genes such as *BRCA1* or *BRCA2* are particularly sensitive to PARP inhibitors, we investigated whether G73-induced degradation of RAD51 could enhance the effects of PARP inhibitors to achieve “fully small-molecules-induced synthetic lethality” in *BRCA2* wild-type cells. To our expectation, the combination of G73 and olaparib significantly potentiated the growth-inhibitory effects of PARP inhibitor olaparib in a dose-dependent manner, leading to a marked increase in cell death (Fig. 4I and Fig. S3F). This synergistic effect was similarly observed for G86 and olaparib (Fig. S3G and S3H). Consistently, coadministration of G73 and olaparib resulted in a more pronounced inhibitory effect on colony formation (Fig. 4J and K).

Given that RAD51 recombinase is critical for HR events mediating genetic information exchange, we examined G73's effect on the cell cycle. Consistent with reported results in *sh*RAD51 and RAD51 inhibitor-treated cells^39^, ^40^, ^41^, G73 significantly increased the percentage of cells in G1 phase relative to the control group. Conversely, RAD51 degradation reduced the proportion of G2/M phases in these prostate cancer cells (Supporting Information Fig. S4A and S4B). This indicates that G73 may arrest the cell cycle at G1 phase, thus inhibiting transition to G2 phase and further suppressing prostate cancer cell proliferation. Given the reported partial dependence of DNA damage-induced G1/S arrest on P21^42^, we investigated the effect of G73 on P21 and its downstream target, CYCLIN D1. The results showed that G73 treatment upregulated both mRNA and protein levels of P21, while concurrently downregulating the cell-cycle protein CYCLIN D1 (Fig. S4C and S4D), implicating the P21-CYCLIN D1 axis in the G73-induced cell cycle blockage.

### 3.3 G73 potentiates PARP inhibitors to suppress tumor growth in xenograft mouse models

**Figure 5:**
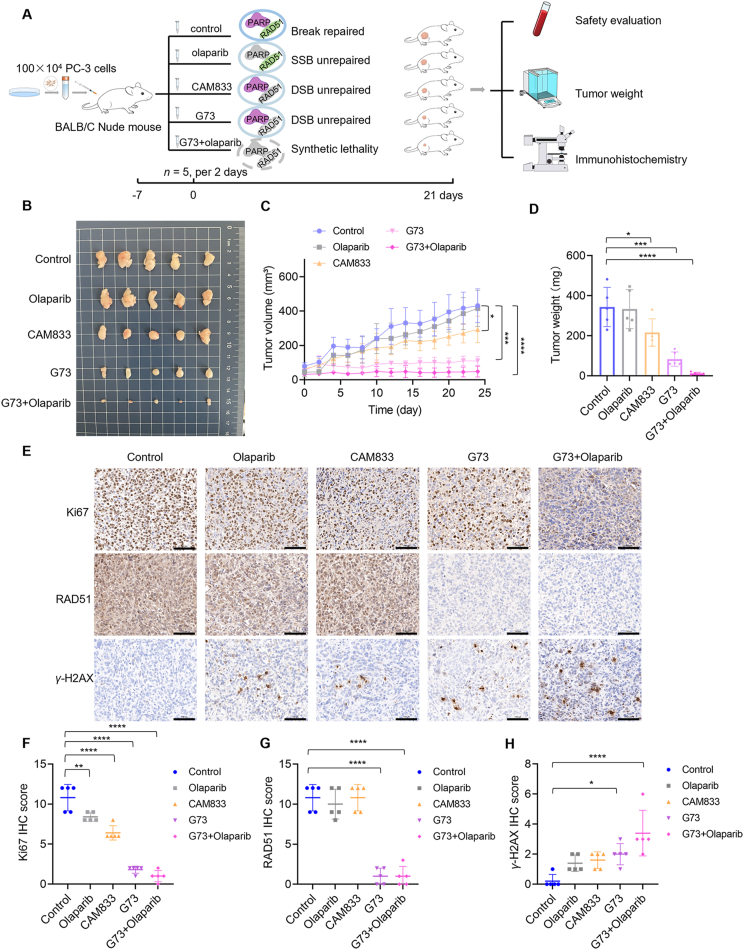
G73 synergizes olaparib to inhibit prostate tumor growth *in vivo.* (A) Schematic of tumor-bearing BALB/c nude mice treated with indicated drugs. 25 BALB/c nude mice were randomly separated into five groups and treated with Control (PBS, *n* = 5), olaparib (50 mg/kg, *n* = 5), CAM833 (50 mg/kg, *n* = 5), G73 (50 mg/kg, *n* = 5) or G73+olaparib (25 mg/kg + 25 mg/kg, *n* = 5) every 2 days. The tumor volume and mice weight of BALB/c nude mice were measured and recorded every 2 days until sacrifice. (B) Photos of PC-3 tumors excised at the end of the experiment after different drug treatments. (C) Tumor growth curves of PC-3 xenografts of BALB/c nude mice in different groups (*n* = 5 per group). Statistical analysis of data was calculated using One-way ANOVA analysis among five groups, error bars indicate ±SD, *n* = 5; ∗*P* < 0.05, ∗∗∗*P* < 0.001, ∗∗∗∗*P* < 0.0001 *vs*. indicated. (D) Average tumor weight excised from each group of mice at the end of drug treatment. The data are presented as the mean ± SD values (*n* = 5). Statistical analysis of data was calculated using One-way ANOVA analysis among five groups; ∗*P* < 0.05, ∗∗∗*P* < 0.001, ∗∗∗∗*P* < 0.0001 *vs*. indicated. (E) Histopathological analysis of the excised tumors from Control, olaparib, CAM833, G73 and G73+olaparib combination groups, using IHC assay for Ki67 staining (tumor cell growth marker), RAD51 staining (RAD51 protein level in tumor cells) and *γ*-H2AX staining. Scale bar = 100 μm. (F‒H) Statistical analysis of IHC scores about Ki67 (F), RAD51 (G) and *γ*-H2AX (H) on PC-3 tumors after different drug treatments. Statistical analysis of data was calculated using One-way ANOVA analysis among groups, *n* = 5; ∗*P* < 0.05, ∗∗*P* < 0.01, ∗∗∗∗*P* < 0.0001 *vs*. indicated.

After confirming the effectiveness of G73 in degrading RAD51 protein and its ability to sensitize prostate cancer cells to PARP inhibitors, we next evaluated its impact on tumor growth in a PC-3 xenograft mouse model. As shown in Fig. S4E, G73 exhibited excellent serum stability at 37 °C with no detectable degradation or precipitation throughout the experimental period, supporting its suitability for further *in vivo* studies. A total of 25 tumor-bearing mice were randomly divided into five treatment groups: PBS, CAM833, olaparib, G73 and the combination of G73 and olaparib, administered every 2 days over 3 weeks (Fig. 5A). As illustrated in Fig. 5B‒D, CAM833 and olaparib alone exhibited minimal tumor growth inhibition, while G73 remarkably enhanced the anti-tumor activity of olaparib in suppressing tumor growth in the PC-3 xenograft model. Immunohistochemical (IHC) analysis further revealed a marked reduction in Ki67 expression, along with a decrease in RAD51 protein levels in the G73 and G73+olaparib combination groups. In addition, the coadministration of G73 and olaparib induced DNA damage, resulting in tumor regression (Fig. 5E‒H and Fig. S4F). These results collectively highlight the potential of the RAD51 degrader G73 as a PARP inhibitor sensitizer, facilitating synthetic lethality as a therapeutic strategy for prostate cancer.

### 3.4 The evaluation of G73's biosafety

**Figure 6:**
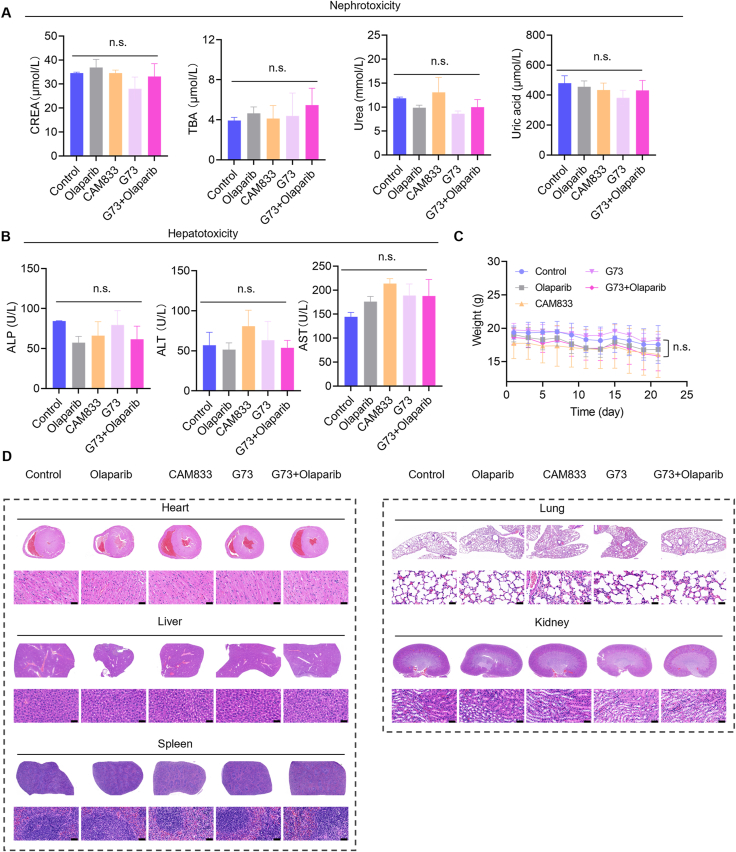
Biosafety analysis of G73. (A, B) Safety characteristics of G73 include nephrotoxicity and hepatotoxicity. 25 healthy C57/BL6 mice were randomly separated into five groups and treated with Control (PBS, *n* = 5), olaparib (50 mg/kg, *n* = 5), CAM833 (50 mg/kg, *n* = 5), G73 (50 mg/kg, *n* = 5) or G73+olaparib (25 mg/kg + 25 mg/kg, *n* = 5) every 2 days. (C) Animal body weights were detected during drug treatment period. The data are presented as the mean ± SD values (*n* = 5). (D) Representative H&E staining photographs of heart, liver, spleen, lung and kidney sections from mice after 21-day treatment of indicated drug. Scale bar = 100 μm; n.s. not significant.

To assess the biosafety of G73, a cohort of 25 healthy C57/BL6 mice was randomly assigned to five groups and treated with intraperitoneal injections of PBS, CAM833, olaparib, G73, or a combination of G73 and olaparib every other day. Following 21 days of administration, the biosafety parameters were evaluated. All tested compounds exhibited minimal impact on kidney function, as indicated by normal levels of creatinine (CREA), total bile acid (TBA), urea, and uric acid (Fig. 6A). Similarly, G73 showed no evidence of hepatotoxicity, with alkaline phosphatase (ALP), aspartate transaminase (ALT) and alanine aminotransferase (AST) levels remaining within normal ranges (Fig. 6B). Furthermore, none of the treatments caused significant changes in body weight over the 21-day period, and hematoxylin and eosin (H&E) staining revealed no noticeable morphological abnormalities in the heart, liver, spleen, or kidneys (Fig. 6C and D). Collectively, these findings suggest that G73 possesses a favorable biosafety profile, supporting its potential as a promising candidate for prostate cancer treatment.

## 4 Discussion

The approval of PARP inhibitors exemplifies the effective application of synthetic lethality in cancer therapy, which spares normal tissues because their HR-proficient cells remain unaffected^43^, ^44^, ^45^, ^46^, ^47^, ^48^, significantly advancing drug development. However, PARP inhibitors have been predominantly restricted to patients with certain gene mutations, who represent only a small portion of all cancer cases. This limitation underscores the need to identify alternative strategies to overcome resistance to PARP inhibitors including combination therapies aimed at further amplifying the anti-tumor effects of PARP inhibitors^49^, thereby broadening their therapeutic applicability.

*RAD51* is a key gene involved in HR, an ATP-driven strand exchange reaction by polymerizing DNA to form a helical filament. Mutations in *RAD51* paralogues are likely to enhance sensitivity to PARP inhibitors^50^. Disrupting the interaction between BRCA2 and RAD51 could mimic the synthetic lethality observed with PARP inhibitors treatment in BRCA2-deficient tumors. However, no RAD51 inhibitors are currently available for clinical use due to challenges in achieving sufficient efficacy. The advent of targeted protein degradation (TPD) as a therapeutic approach offers the potential to tackle disease-causing proteins that have historically been highly challenging to target with conventional small molecules^51^^,^^52^ and thus opens new possibilities for developing novel molecules that target RAD51 with enhanced efficacy.

In this study, we present the identification of the RAD51 degrader G73, which reduces RAD51 protein levels at sub-micromolar concentrations. It induced the degradation of RAD51 in a dose- and time-dependent manner, impairing the activity of HR pathway, as evidenced by the increased formation of *γ*-H2AX. The loss of RAD51 protein mediated by G73 mimics the effects of genetic *RAD51* deletion, a clinically identified HR-deficient phenotype, thereby sensitizing HR-proficient cancers to PARP inhibitors. As a result, significant tumor regression was observed in mice treated with a combination of G73 and olaparib. Furthermore, G73 exhibited minimal toxicity, as no notable changes were detected in blood biochemical parameters or tissue histomorphology.

**Figure 7:**
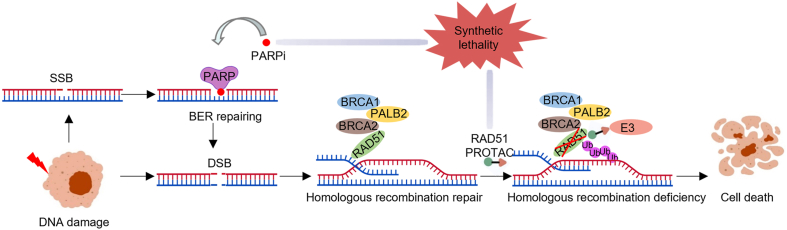
Schematic diagram showing the synthetic lethality between PARP inhibitors and RAD51-targeting degraders to induce cancer cell death.

PARP inhibitors are increasingly utilized in clinical settings and are now being applied to indications beyond breast and ovarian cancers. Researches involving patients with ovarian^53^^,^^54^ and prostate cancers^55^ have shown that those with *BRCA1/2*-wild-type tumors harbouring deleterious mutations in other DNA repair genes—such as *PALB2*, *ATM*, *CHEK2*, *CDK12*, *FANCA*, *RAD54L* and *BRIP1*—might benefit from PARP inhibitors. Mutations in other key HR-related genes, such as *RAD51* paralogues, are also likely to confer sensitivity to PARP inhibitors^50^. Our findings provide proof-of-concept that PROTAC-mediated degradation of RAD51, an HR-related protein, can mimic a clinically identified HR-deficient phenotype, thereby holding promise for broadening the clinical application of PARP inhibitors and addressing acquired resistance to these therapies (Fig. 7). These results highlight a unique opportunity to restore the anti-tumor efficacy of PARP inhibitors, and this approach could be extended to target other HR-related genes such as *ATM*, *CHEK2*, *CDK12*, *FANCA*, *RAD54L* and *BRIP1*.

## Author contributions

Lei Li conceived and directed the project. Yanlin Jian designed, synthesized and characterized the compounds. Yibo Gao, Tianyang Zhou, Shan Xu, and Bin Wang contributed to the *in vitro* biology experiments and data analysis. Yanlin Jian and Lei Li wrote the manuscript. Yizeng Fan, Jian Ma, Yang Gao, Jing Liu, Bohan Ma reviewed and edited the manuscript. All authors read and approved of the final manuscript.

## Conflicts of interest

The authors declare no conflicts of interest.

## Appendix A Supporting information

The following is the Supporting Information to this article.
