# Supplementary material for: RAD51-targeting small molecule degrader sensitizes BRCA-proficient prostate cancer cells to PARP inhibitors via synthetic lethality
Source: Acta Pharm Sin B. 2026 Mar 19;16(6):3907–19. doi: 10.1016/j.apsb.2026.03.029 (PMC13304771; doi:10.1016/j.apsb.2026.03.029)
Supplement: Multimedia component 1 [file mmc1.pdf]

Supporting Information for

ORIGINAL ARTICLE

RAD51-targeting small molecule degrader sensitizes *BRCA*-proficient prostate cancer cells to PARP inhibitors *via* synthetic lethality

Yanlin Jian†, Yibo Gao†, Tianyang Zhou†, Shan Xu, Bin Wang, Yizeng Fan, Jian Ma, Yang Gao, Jing Liu, Bohan Ma*, Lei Li*

Department of Urology, the First Affiliated Hospital of Xi'an Jiaotong University, Xi'an 710061, China

Received 4 August 2025; received in revised form 9 October 2025; accepted 17 November 2025

*Corresponding authors.

E-mail addresses: lilydr@163.com (Lei Li), bohanma1222@gmail.com (Bohan Ma).

†These authors made equal contributions to this work.

Table of contents

Figure S1 presentative scaffolds of RAD51 small-molecule inhibitors…….……3

Figure S2 The development of RAD51 degraders.……………………….……4

Figure S3 Anticancer effect of RAD51 degraders……………………..…5

Figure S4 The stability of G73 and its effect on cell cycle distribution…………6

Cell cycle flow cytometry……………………..……...…..………………………7

P21 and *CYCLIN D1* mRNA level detection by RT-qPCR…………………… …7

Synthesis details and characterization of compounds…………………...………7

Chemistry general procedures………………………………………………………7

Scheme S1 Synthesis of **7**………………………………………..……...…8

Scheme S2 Synthesis of **8**, **9–14**, **15–22**……………………………..……….11

References……………………….……………..………………………......26

Spectra of active compound G73…………………………………….………..27

1. Supporting figures

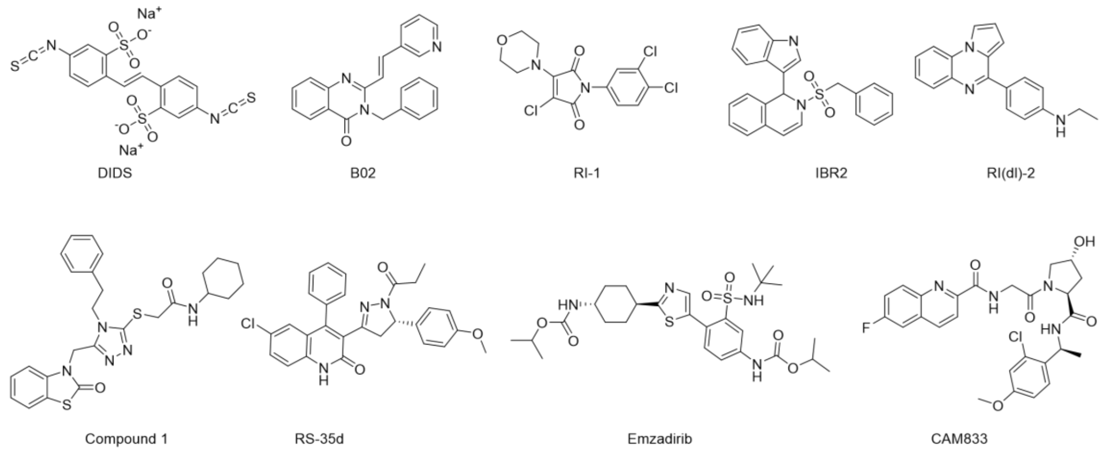

Figure S1 Representative scaffolds of RAD51 small-molecule inhibitors.

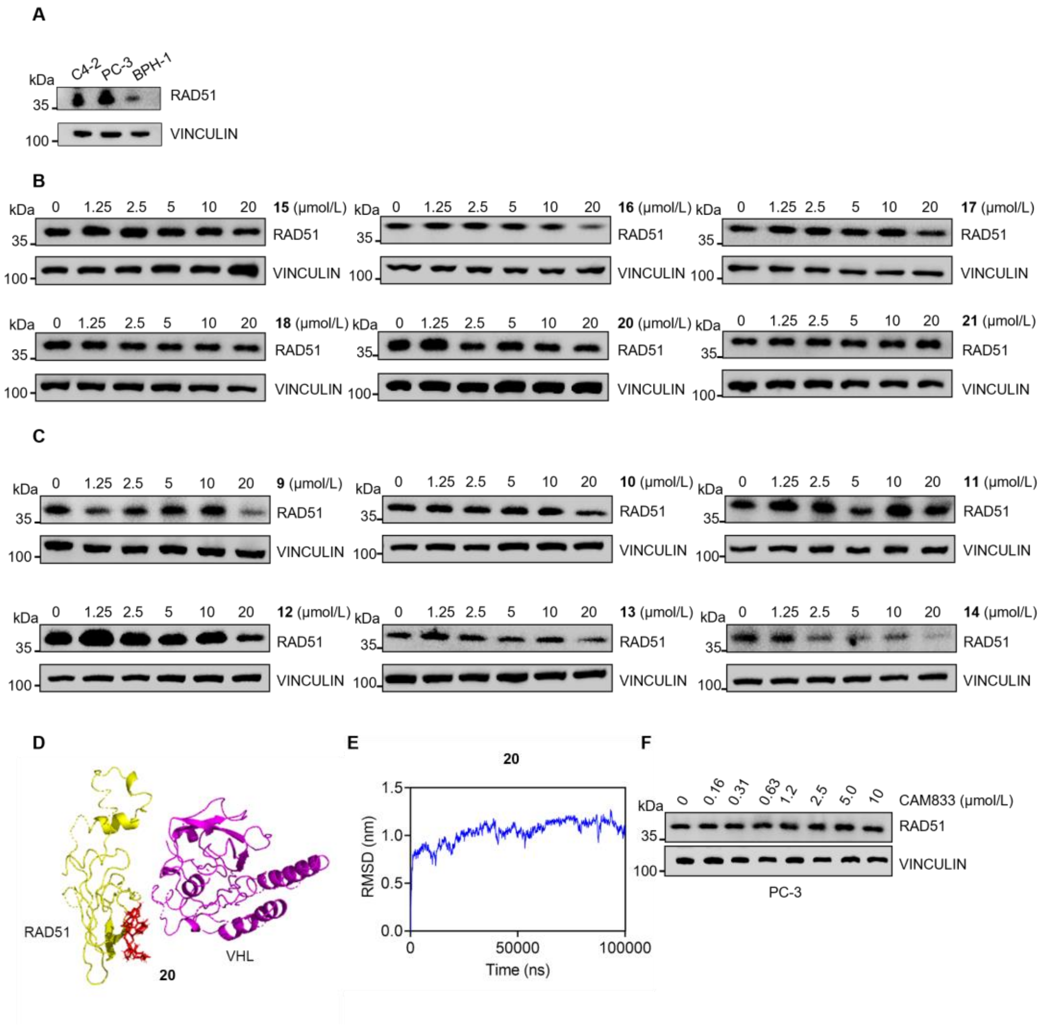

Figure S2 The development of RAD51 degraders. (A) The expression of RAD51 protein in different cell lines. (B) Immunoblot analysis of cells treated with increasing concentrations of VHL-based RAD51 degraders. (C) Immunoblot analysis of cells treated with increasing concentrations of CRBN-based RAD51 degraders. (D) The predicted binding mode of the RAD51: **20**: VHL ternary complex (yellow: RAD51, magenta: VHL). (E) The RMSD curve of the ternary complex induced by compound **20**. (F) Immunoblot analysis of cells treated with increasing concentrations of CAM833.

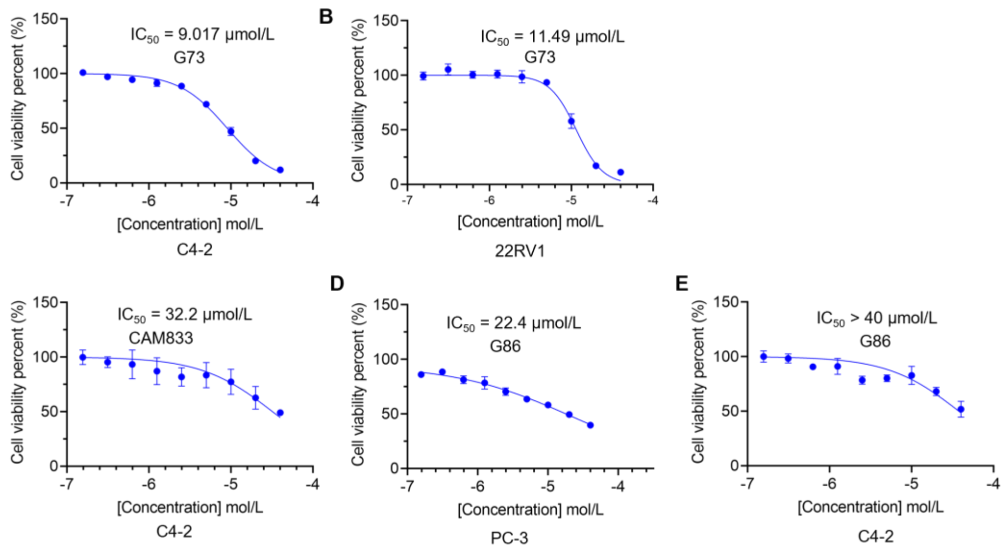

F

Olaparib (μmol/L)	G73 (μmol/L)	
	2.5	5
0	1.03	1.18
0.39	0.96	1.06
0.78	0.89	0.96
1.56	0.86	0.98
3.13	0.82	0.87
6.25	0.74	0.77
12.5	0.75	0.77
25	0.66	0.70
50	0.53	0.55
100	0.49	0.49

G

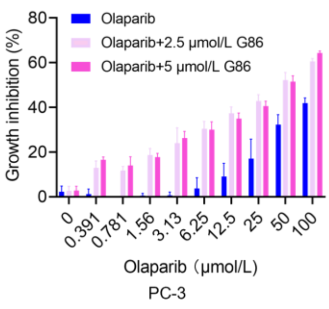

H

Olaparib (μmol/L)	G86 (μmol/L)	
	2.5	5
0	1.15	1.10
0.39	0.96	0.92
0.78	0.92	0.90
1.56	0.86	0.87
3.13	0.82	0.79
6.25	0.78	0.79
12.5	0.77	0.80
25	0.77	0.80
50	0.79	0.80
100	0.76	0.69

Figure S3 Anticancer effect of RAD51 degraders. (A–E) Cell viability of prostate cancer cells was detected after 48 h treatment with G73, CAM833 or G86. Data are presented as mean ± SD (n = 3). (F) Calculation of combination index of G73, synergism is indicated by a result < 0.8. (G) Cell viability of PC-3 cells after 48 h treatment with varying concentrations of indicated drug. Data are presented as mean ± SD (n = 3). (H) Calculation of combination index of G86, synergism is indicated by a result < 0.8.

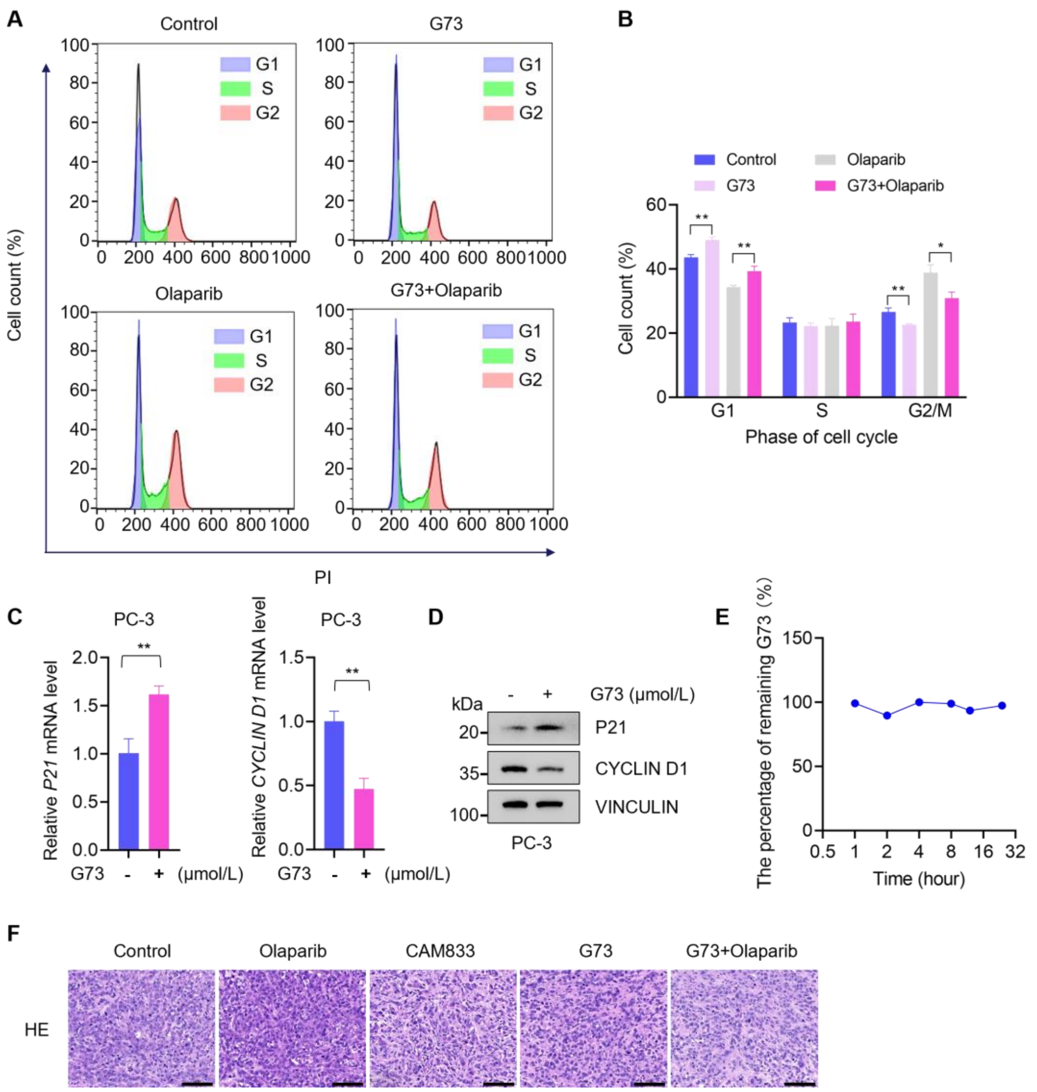

Figure S4 The stability of G73 and its effect on cell cycle distribution. (A) Flow cytometric analysis of the distribution of cell cycle phases of PC-3 cells treated with indicated drug for 24 h. G73: 5 μmol/L; Olaparib: 10 μmol/L. (B) Quantitative analysis of the cell cycle distribution. Data are presented as mean ± SD (n = 3). Unpaired two-tailed Student's t tests, *P < 0.05, **P < 0.01 *vs.* indicated. (C) RT-qPCR analysis of *P21* and *CYCLIN D1* mRNA levels in PC-3 cells treated with G73 (10 μmol/L) for 24 h. Data are presented as mean ± SD (n = 3). Unpaired two-tailed Student's t tests, **P < 0.01 *vs.* indicated. (D) Immunoblot analysis of P21 and CYCLIN D1 protein levels in PC-3 cells treated with G73 (10 μmol/L) for 24 h. (E) Stability analysis of G73 in serum as detected by LC-MS. (F) Histopathological analysis of the excised tumors. Scale bar=100 μm.

2. Supporting Information: Materials and methods

2.1. Cell cycle flow cytometry

PC-3 cells were seeded at a density of 2×10^5 on 6 cm dishes. After 24-h treatment with indicated drug, cells were collected by centrifugation (1000 rpm, 2 min), washed with PBS, and fixed in 0.5 mL ice-cold 70% ethanol at 4 °C for 30 min. Fixed cells were then washed three times with PBS and stained with PBS containing 50 μg/mL propidium iodide (PI) and 200 μg/mL RNase A at 37 °C for 30 min in the dark. Finally, cells were filtered through a 200-mesh strainer and analysed by flow cytometry to determine cell cycle distribution.

2.2. P21 and CYCLIN D1 mRNA level detection by RT-qPCR

Total RNA of PC-3 cells was extracted (RNAfast200 Kit, Fastagen) and reverse transcribed (PrimeScript RT Master Mix, Takara). RT-qPCR was conducted on a Bio-Rad CFX96 system using SYBR Green Master qPCR Mix (ABclonal). Data were normalized to 18S rRNA and analyzed by the $2^{-\Delta\Delta Ct}$ method. Primer sequences were listed as follows: P21 (forward primer 5′-TGTCCGTCAGAACCCATGC-3′; reverse primer 5′-AAAGTCGAAGTTCCATCGCTC-3′); *CYCLIN* D1 (forward primer 5′-GCTGCGAAGTGGAAACCATC-3′; reverse primer 5′-CCTCCTTCTGCACACATTTGAA-3′).

2.3. Synthesis details and characterization of compounds

Chemistry general procedures

All reagents and solvents were purchased from standard commercial sources and were of analytical grade. All synthetic compounds described in this study were checked with analytical TLC (Machery-Nagel precoated F254 aluminium plates), visualized under UV light at 254 nm, and purified by column chromatography (CC) on a Reveleris X2 (Grace) automated flash unit. NMR data were recorded on Bruker Avance Neo® 400/100 MHz or a Bruker Avance Neo® 600/150 MHz spectrometer at 298.15 K using residual solvent signal as reference. Confirmation of compound structure was conducted with ^1H, ^{13}C and HSQC NMR spectrometry. Additionally, high resolution mass spectrometry was performed on a Waters LCT Premier XE™ time of flight (TOF) mass spectrometer equipped with a standard electrospray ionization (ESI) and modular LockSpray™ interface. The purity (95%) of the tested compounds was determined by

LC–MS analysis (Waters AutoPurification system: a Waters Cortecs C18 column (2.7 μm, 100 mm × 4.6 mm); a gradient system of formic acid in H_2O (0.2%, *v/v*)/MeCN; a flow rate of 0.30 mL/min; a gradient of 95:5 to 0:100 in 15 min).

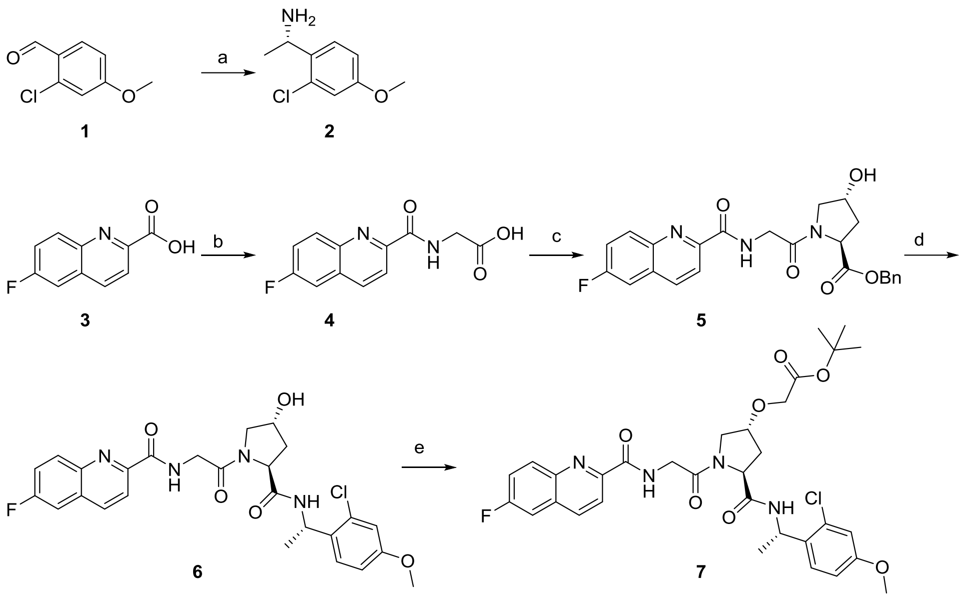

Scheme S1 Synthesis of **7**[α]. [α]Reagents and conditions: (a) (i) (*R*)-(+)-*t*-butanesulfinamide, $Ti(OEt)_4$, tetrahydrofuran (THF), reflux; (ii) MeMgBr, CH_2Cl_2; (iii) 4 mol/L HCl, 1,4-dioxane; (b) (i) Glycine methyl ester hydrochloride, benzotriazol-1-yl-oxytripyrrolidino-phosphonium hexafluorophosphate (PyBOP), *N*,*N*-diisopropylethylamine (DIPEA), CH_2Cl_2; (ii) LiOH, MeOH/THF (1:1, *v/v*); (c) Benzyl (2*S*,4*R*)-4-hydroxypyrrolidine-2-carboxylate, PyBOP, DIPEA, CH_2Cl_2; (d) (i) Pd/C, H_2, MeOH; (ii) (*S*)-1-(2-chloro-4-methoxyphenyl)ethan-1-amine, *O*-benzotriazole-*N*,*N*,*N'*,*N'*-tetramethyl-uronium-hexafluorophosphate (HBTU), DIPEA, CH_2Cl_2; (e) Bromoacetic acid *tert*-butyl ester, NaH, THF.

(*S*)-1-(2-chloro-4-methoxyphenyl)ethan-1-amine (2) To a solution of 2-chloro-4-methoxybenzyaldehyde (**1**) (100 mg, 0.586 mmol) and (*R*)-(+)-*t*-butanesulfinamide (71 mg, 0.586 mmol) in THF (3 mL) was added $Ti(OEt)_4$ (294 mg, 1.29 mmol). The reaction mixture was refluxed for overnight under N_2 atmosphere. After cooling to room temperature, the mixture was diluted with ethyl acetate (25 mL) and washed with brine. The mixture was filtered through celite and separated. The collected organic layer was dried over Na_2SO_4 and concentrated to give the sulfinimide intermediate. The sulfinimide intermediate was dissolved in CH_2Cl_2 (1 mL) and the mixture was cooled

to –20 °C, followed by dropwise addition of MeMgBr (342 μL, 1.08 mmol). The reaction was warmed to room temperature and stirred for overnight. The resulting mixture was quenched with saturated aq. NH_4Cl (2 mL), and extracted with CH_2Cl_2. The combined organic layer was dried over Na_2SO_4, concentrated and purified with flash column to give the sulfinamine. The obtained sulfinamine was dissolved in 1,4-dioxane (1 mL), followed by slow addition of 4 mol/L HCl (in 1,4-dioxane, 320 μL). The reaction was stirred at room temperature for 1 h and concentrated *in vacuo*. The residue was dissolved in water, neutralized with aq. 4 mol/L NaOH to pH 14 and extracted with CH_2Cl_2. The combined organic layer was dried over Na_2SO_4 and concentrated to give the title compound[1].

6-Fluoroquinoline-2-carbonyl)glycine (4) To a solution of 6-fluoroquinoline-2-carboxylic acid (**3**) (100 mg, 0.53 mmol), glycine methyl ester hydrochloride (73 mg, 0.57 mmol) and DIPEA (212 μL) in CH_2Cl_2 at 0 °C was added PyBOP (300 mg, 0.57 mmol). The reaction mixture was warmed to room temperature and stirred for 16 h. The reaction mixture was diluted with CH_2Cl_2, washed with sat. NaCl. The combined organic layer was dried over Na_2SO_4, concentrated and purified with flash column to give the methyl ester. The ester was dissolved in a MeOH/THF/water mix (1:2:1, 2 mL), followed by addition of LiOH (35.9 mg, 1.5 mmol), and the resulting mixture was stirred at room temperature for 3 h. The mixture was concentrated *in vacuo*, and the residue was acidified with 3 mol/L HCl to pH 2. The suspension was extracted with ethyl acetate, and the combined organic layer was concentrated to give the title compound without further purification[1].

Benzyl(2*S*,4*R*)-1-((6-fluoroquinoline-2-carbonyl)glycyl)-4-hydroxypyrrolidine-2-carboxylate (5) Compound **5** was synthesized following the same procedure for compound **4**. Compound **4** (80 mg, 0.32 mmol), benzyl (2*S*,4*R*)-4-hydroxypyrrolidine-2-carboxylate(93 mg, 0.36 mmol), DIPEA (213 μL) and PyBOP (171 mg, 0.36 mmol) in CH_2Cl_2 gave the title compound[1].

N-(2-((2*S*,4*R*)-2-(((*S*)-1-(2-chloro-4-methoxyphenyl)ethyl)carbamoyl)-4-hydroxypyrrolidin-1-yl)-2-oxoethyl)-6-fluoroquinoline-2-carboxamide (6) To a solution of compound **5** (44 mg, 0.098 mmol) in MeOH (1 mL) was added Pd/C (10%

wt. Pd, 5 mg). The mixture was stirred under H_2 balloon overnight and filtered through celite. The resulting mixture was concentrated and redissolved in CH_2Cl_2. Then, (*S*)-1-(2-chloro-4-methoxyphenyl)ethan-1-amine (**2**, 15.3 mg, 0.083 mmol), DIPEA (96.8 μL), and HATU (38.7 mg, 0.102 mmol) were added, and the reaction mixture was stirred at room temperature for 3 h. The mixture was washed with sat. NaCl, extracted with CH_2Cl_2, dried over Na_2SO_4. The concentrated residue was purified with flash column to give the title compound[1].

***Tert*-butyl 2-(((3*R*,5*S*)-5-(((*S*)-1-(2-chloro-4-methoxyphenyl)ethyl)carbamoyl)-1-((6-fluoroquinoline-2-carbonyl)glycyl)pyrrolidin-3-yl)oxy)acetate (7)** To a suspension of NaH (60% dispersion in oil, 33 mg, 0.828 mmol), *tert*-butyl bromoacetate (296 μL) and *n*-$Bu_4N^+Cl^-$ (15.4 mg, 0.055 mmol) in THF at 0 °C under a N_2 atmosphere was added compound **6** (290 mg, 0.552 mmol). The mixture was warmed to room temperature and stirred overnight. Then, the reaction was quenched with sat. NH_4Cl, washed with sat. NaCl and dried over Na_2SO_4. The concentrated residue was purified with flash column to give the title compound.

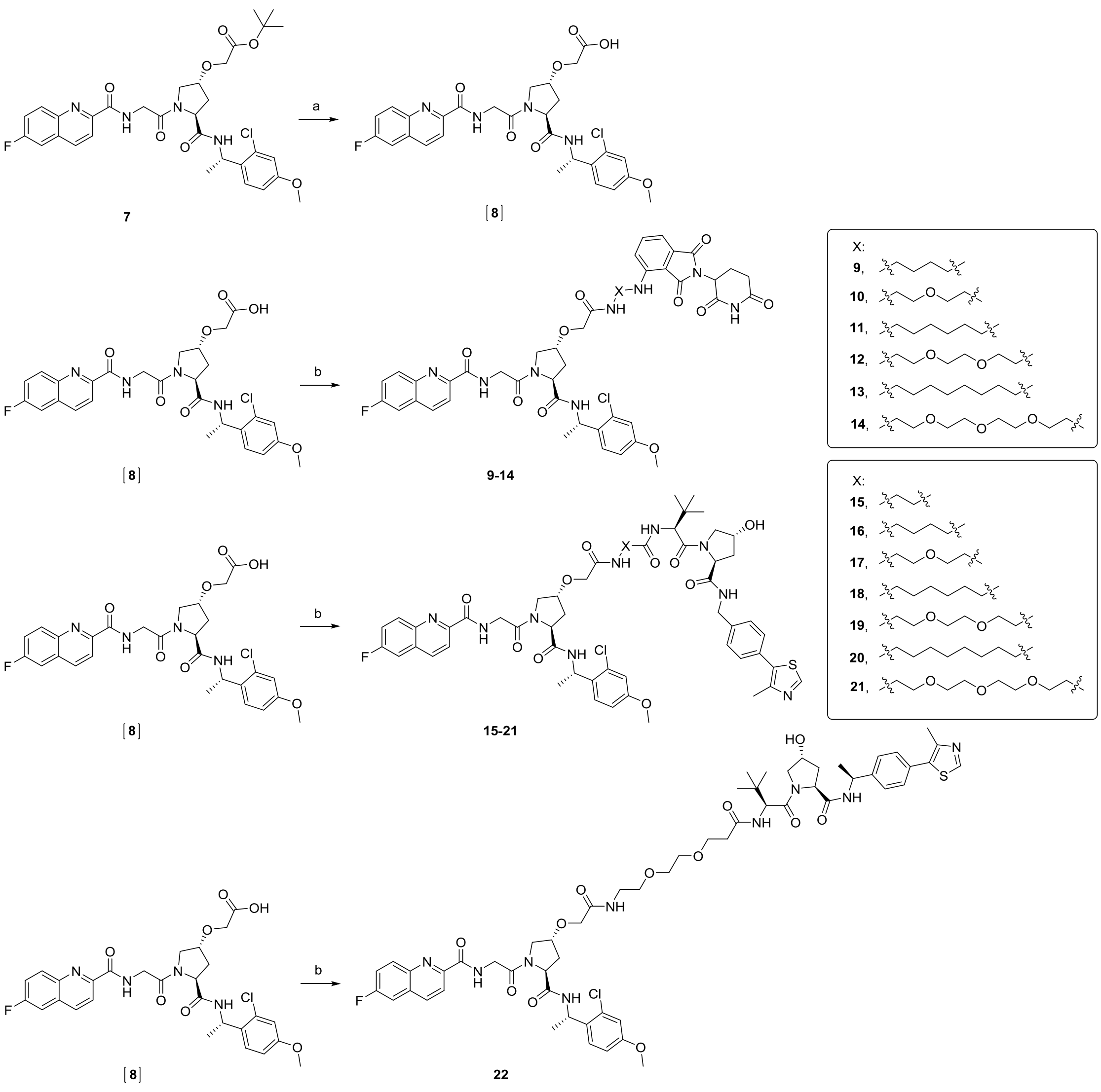

Scheme S2 Synthesis of **8**, **9–14**, **15–22**$^{\alpha}$. $^{\alpha}$Reagents and conditions: (a) Trifluoroacetic acid (TFA), CH_2Cl_2; (b) DIPEA, 2-(7-azabenzotriazol-1-yl)-*N*,*N*,*N′*,*N′*-tetramethyluronium hexafluorophosphate (HATU), CRBN/VHL ligand-based linker, DMF.

2-(((3*S*,5*S*)-5-(((*S*)-1-(2-chloro-4-methoxyphenyl)ethyl)carbamoyl)-1-((6-fluoroquinoline-2-carbonyl)glycyl)pyrrolidin-3-yl)oxy)acetic acid (8) To a solution of compound **7** (80 mg, 0.151 mmol) in CH_2Cl_2 (1 mL) was added TFA (2 mL). The mixture was stirred at room temperature for 3 h. The mixture was concentrated *in vacuo* to give the title compound used for next step without purification.

***N*-(2-((2*S*,4*S*)-2-(((*S*)-1-(2-chloro-4-methoxyphenyl)ethyl)carbamoyl)-4-(2-((4-((2-(2,6-dioxopiperidin-3-yl)-1,3-dioxoisoindolin-4-yl)amino)butyl)amino)-2-**

oxoethoxy)pyrrolidin-1-yl)-2-oxoethyl)-6-fluoroquinoline-2-carboxamide (9) 2-(2,6-Dioxopiperidin-3-yl)-4-fluoroisoindoline-1,3-dione (100 mg, 0.362 mmol) was dissolved in DMF (1 mL). Then, *tert*-butyl (4-aminobutyl)carbamate (75 mg, 0.398 mmol) and DIPEA (252 μL) were added, and the reaction mixture was stirred at 90 °C overnight. The mixture was washed with sat. NaCl, extracted with ethyl acetate, dried over Na_2SO_4. The concentrated residue was purified with flash column to give *tert*-butyl (4-((2-(2,6-dioxopiperidin-3-yl)-1,3-dioxoisoindolin-4-yl)amino)butyl)carbamate (112 mg, 70%). The resulting product was dissolved in CH_2Cl_2 (1 mL), followed by addition of TFA (2 mL). The mixture was stirred at room temperature for 3 h and concentrated *in vacuo*. The residue was redissolved in DMF (2 mL). Then, compound **8** (112 mg, 0.191 mmol), DIPEA (102 μL), and HATU (87 mg, 0.229 mmol) were added, and the reaction was stirred at room temperature for 3 h. The mixture was washed with sat. NaCl, extracted with ethyl acetate, dried over Na_2SO_4. The concentrated residue was purified with flash column to give the title compound (132 mg, 76%). HRMS calculated for $C_{45}H_{46}ClFN_8O_{10}$, 935.29012 (M+Na^+), found 935.29331. ^{1}H NMR (600 MHz, DMSO-d_6) δ ppm 1.30 (d, *J*=7.15 Hz, 2.1 H), 1.39 (d, *J*=6.97 Hz, 0.9 H), 1.46–1.60 (m, 4 H), 1.80–1.86 (m, 0.7 H), 1.98–2.05 (m, 1.3 H), 2.33 (t, *J*=9.81 Hz, 0.7 H), 2.47–2.48 (m, 0.3 H), 2.53 (br. s., 1 H), 2.58 (d, *J*=17.42 Hz, 1 H), 2.83–2.93 (m, 1 H), 3.09–3.19 (m, 2 H), 3.26–3.31 (m, 3 H), 3.44–3.46 (m, 0.3 H), 3.68–3.71 (s, 3 H), 3.74 (s, 0.7 H), 3.79–3.85 (m, 1 H), 3.86 (s, 0.3 H), 3.88–3.89 (m, 1 H), 4.11–4.23 (m, 2 H), 4.31 (dd, *J*=17.15, 5.23 Hz, 0.7 H), 4.41 (t, *J*=7.79 Hz, 0.7 H), 4.62–4.64 (m, 0.3 H), 5.04 (dd, *J*=12.84, 5.32 Hz, 1 H), 5.11 (quin, *J*=7.15 Hz, 0.8 H), 5.20–5.23 (m, 0.2 H), 6.50–6.60 (m, 1 H), 6.84 (dd, *J*=8.71, 2.48 Hz, 0.7 H), 6.93–6.94 (m, 0.3 H), 6.95 (d, *J*=2.38 Hz, 0.7 H), 6.97–7.03 (m, 1.3 H), 7.04–7.06 (m, 0.3 H), 7.09 (d, *J*=8.62 Hz, 0.7 H), 7.30 (d, *J*=8.62 Hz, 0.7 H), 7.36–7.37 (m, 0.3 H), 7.51–7.58 (m, 1 H), 7.73–7.85 (m, 2 H), 7.89–7.96 (m, 1 H), 8.17–8.24 (m, 2 H), 8.43 (d, *J*=7.52 Hz, 0.7 H), 8.55–8.60 (m, 1 H), 8.81–8.83 (m, 0.3 H), 8.93–9.00 (m, 1 H), 11.09 (s, 1 H). ^{13}C NMR (151 MHz, DMSO-d_6) δ ppm 21.3 (1 C), 22.2 (1 C), 26.2 (1 C), 26.7 (1 C), 31.0 (1 C), 34.7 (1 C), 37.9 (1 C), 41.5 (1 C), 44.9 (1 C), 48.5 (1 C), 51.3 (1 C), 55.5 (2 C), 58.6 (1 C), 67.9 (1 C), 78.0 (1 C), 109.0 (1 C), 110.4 (1 C), 111.3 (1 C), 113.4 (1 C), 114.3 (1 C), 117.2 (1 C), 119.3 (1 C), 120.9 (1 C), 127.4 (1 C), 129.8

(1 C), 131.8 (1 C), 132.2 (1 C), 133.9 (1 C), 136.3 (1 C), 137.6 (1 C), 143.2 (1 C), 146.4 (1 C), 149.2 (1 C), 158.5 (1 C), 158.9 (1 C), 159.8 (0.5 C), 161.4 (0.5 C), 163.7 (1 C), 166.8 (1 C), 167.3 (1 C), 168.8 (1 C), 168.9 (1 C), 170.0 (1 C), 170.1 (1 C), 172.8 (1 C).

Compounds 10–14 was synthesized following the same procedure for compound 9.

***N*-(2-((2*S*,4*S*)-2-(((*S*)-1-(2-chloro-4-methoxyphenyl)ethyl)carbamoyl)-4-(2-((2-(2-((2-(2,6-dioxopiperidin-3-yl)-1,3-dioxoisoindolin-4-yl)amino)ethoxy)ethyl)amino)-2-oxoethoxy)pyrrolidin-1-yl)-2-oxoethyl)-6-fluoroquinoline-2-carboxamide (10)** 2-(2,6-Dioxopiperidin-3-yl)-4-fluoroisoindoline-1,3-dione (100 mg, 0.362 mmol), *tert*-butyl (2-(2-aminoethoxy)ethyl)carbamate (86 mg, 0.398 mmol) and DIPEA (252 μL) in DMF (1 mL) gave *tert*-butyl (2-(2-((2-(2,6-dioxopiperidin-3-yl)-1,3-dioxoisoindolin-4 yl)amino)ethoxy)ethyl)carbamate (149 mg, 81%). Then, the deBoc product, compound **8** (146 mg, 0.249 mmol), DIPEA (133 μL), and HATU (113 mg, 0.300 mmol) in DMF (2 mL) gave compound **10** (163 mg, 71%). HRMS calculated for $C_{45}H_{46}ClFN_8O_{11}$, 951.28512 (M+Na$^+$), found 951.28640. ^{1}H NMR (600 MHz, DMSO-d_6) δ ppm 1.30 (d, *J*=6.60 Hz, 2.1 H), 1.38 (br. s., 0.9 H), 1.80–1.86 (m, 0.6 H), 1.95–1.97 (m, 0.4 H), 1.99–2.02 (m, 1 H), 2.29–2.36 (m, 0.7 H), 2.52–2.62 (m, 2.3 H), 2.83–2.92 (m, 1 H), 3.26–3.31 (m, 2 H), 3.41–3.43 (m, 0.3 H), 3.48–3.51 (m, 4 H), 3.60–3.62 (m, 2 H), 3.66–3.69 (m, 0.7 H), 3.70–3.72 (m, 2.3 H), 3.74–3.76 (m, 0.7 H), 3.78–3.82 (m, 1 H), 3.84–3.88 (m, 0.3 H), 3.88–3.99 (m, 2 H), 4.11–4.22 (m, 2 H), 4.31 (dd, *J*=17.06, 5.32 Hz, 0.7 H), 4.39–4.47 (m, 0.7 H), 4.60–4.64 (m, 0.3 H), 5.05 (dd, *J*=12.56, 4.86 Hz, 1 H), 5.10 (quin, *J*=7.29 Hz, 0.8 H), 5.18–5.24 (m, 0.2 H), 6.59 (br. s., 1 H), 6.84 (dd, *J*=8.71, 2.48 Hz, 0.7 H), 6.92–6.93 (m, 0.3 H), 6.95 (d, *J*=2.38 Hz, 0.7 H), 6.99–7.00 (m, 0.3 H), 7.02 (d, *J*=6.97 Hz, 1 H), 7.13 (d, *J*=8.62 Hz, 1 H), 7.30 (d, *J*=8.62 Hz, 0.7 H), 7.36–7.39 (m ,0.3 H), 7.53–7.59 (m, 1 H), 7.71–7.75 (m, 0.3 H), 7.77–7.84 (m, 1.7 H), 7.90–7.95 (m, 1 H), 8.16–8.20 (m, 2 H), 8.43 (dd, *J*=7.34, 3.67 Hz, 0.7 H), 8.55–8.60 (m, 1 H), 8.79–8.84 (m, 0.3 H), 8.94–9.00 (m, 1 H), 11.10 (s, 1 H). ^{13}C NMR (151 MHz, DMSO-d_6) δ ppm 21.3 (1 C), 22.1 (1 C), 31.3 (1 C), 34.7 (1 C), 38.0 (1 C), 41.6 (1 C), 44.9 (1 C), 48.5 (1 C), 51.3 (1 C), 55.5 (2 C), 58.6 (1 C), 67.9 (1 C), 68.6 (1 C), 68.8 (1 C), 78.0 (1 C), 109.2 (1 C), 110.7 (1 C), 111.3 (1 C), 113.4 (1 C), 114.3 (1 C), 117.4 (1 C), 119.3 (1 C), 121.0 (1 C), 127.4 (1 C), 129.7 (1 C), 131.8 (1 C), 132.1 (1

C), 133.9 (1 C), 136.2 (1 C), 137.7 (1 C), 143.2 (1 C), 146.4 (1 C), 149.2 (1 C), 158.5 (1 C), 158.8 (1 C), 159.8 (0.5 C), 161.4 (0.5 C), 163.6 (1 C), 166.8 (1 C), 167.3 (1 C), 168.9 (1 C), 169.0 (1 C), 170.0 (1 C), 170.1 (1 C), 172.8 (1 C).

***N*-(2-((2*S*,4*S*)-2-(((*S*)-1-(2-chloro-4-methoxyphenyl)ethyl)carbamoyl)-4-(2-((6-((2-(2,6-dioxopiperidin-3-yl)-1,3-dioxoisoindolin-4-yl)amino)hexyl)amino)-2-oxoethoxy)pyrrolidin-1-yl)-2-oxoethyl)-6-fluoroquinoline-2-carboxamide (11)** 2-(2,6-Dioxopiperidin-3-yl)-4-fluoroisoindoline-1,3-dione (100 mg, 0.362 mmol), *tert*-butyl (6-aminohexyl)carbamate (81 mg, 0.398 mmol) and DIPEA (252 μL) in DMF (1 mL) gave *tert*-butyl (6-((2-(2,6-dioxopiperidin-3-yl)-1,3-dioxoisoindolin-4-yl)amino)hexyl)carbamate (133 mg, 78%). Then, the deBoc product, compound **8** (138 mg, 0.235 mmol), DIPEA (126 μL), and HATU (107 mg, 0.282 mmol) in DMF (2 mL) gave compound **11** (155 mg, 70%). HRMS calculated for $C_{47}H_{50}ClFN_8O_{10}$, 963.32142 ($M+Na^+$), found 963.32306. ^{1}H NMR (600 MHz, DMSO-d_6) δ ppm 1.27–1.35 (m, 6 H), 1.38 (d, *J*=6.97 Hz, 1 H), 1.39–1.46 (m, 2 H), 1.48–1.58 (m, 2 H), 1.82 (ddd, *J*=13.20, 7.79, 4.86 Hz, 0.7 H), 1.98–2.04 (m, 1.3 H), 2.34 (t, *J*=9.81 Hz, 0.7 H), 2.45–2.49 (m, 0.3 H), 2.51–2.55 (m, 1 H), 2.58 (d, *J*=17.24 Hz, 1 H), 2.86 (ddd, *J*=17.29, 13.98, 5.41 Hz, 1 H), 3.06–3.14 (m, 2 H), 3.24 (q, *J*=6.60 Hz, 1 H), 3.52–3.55 (m, 2.3 H), 3.66–3.71 (m, 3 H), 3.73 (s, 0.7 H), 3.79–3.85 (m, 1 H), 3.88 (s, 0.3 H), 3.90 (s, 1 H), 4.10–4.22 (m, 2 H), 4.27–4.33 (m, 0.7 H), 4.41 (t, *J*=7.89 Hz, 0.7 H), 4.61–4.65 (m, 0.3 H), 5.03 (dd, *J*=12.84, 5.50 Hz, 1 H), 5.09 (t, *J*=7.15 Hz, 0.8 H), 5.17–5.23 (m, 0.2 H), 6.43–6.51 (m, 1 H), 6.83 (dd, *J*=8.80, 2.57 Hz, 0.7 H), 6.91–6.93 (m, 0.3 H), 6.94 (d, *J*=2.57 Hz, 0.7 H), 6.97–7.00 (m, 1.3 H), 7.01–7.03 (m, 0.3 H), 7.04 (d, *J*=8.62 Hz, 0.7 H), 7.29 (d, *J*=8.62 Hz, 0.7 H), 7.34–7.38 (0.3 H), 7.52–7.58 (m, 1 H), 7.68–7.73 (m, 0.3 H), 7.75–7.83 (m, 1.7 H), 7.87–7.92 (m, 1 H), 8.15–8.23 (m, 2 H), 8.46 (d, *J*=7.52 Hz, 0.7 H), 8.53–8.59 (m, 1 H), 8.82–8.84 (m, 0.3 H), 8.98 (t, *J*=5.14 Hz, 1 H), 11.09 (s, 1 H). ^{13}C NMR (151 MHz, DMSO-d_6) δ ppm 21.4 (1 C), 22.3 (1 C), 26.2 (1 C), 26.3 (1 C), 28.7 (1 C), 29.3 (1 C), 31.1 (1 C), 34.8 (1 C), 38.3 (1 C), 41.9 (1 C), 45.1 (1 C), 48.7 (1 C), 51.5 (1 C), 55.6 (1 C), 55.7 (1 C), 58.7 (1 C), 68.0 (1 C), 78.1 (1 C), 109.1 (1 C), 110.6 (1 C), 111.4 (1 C), 113.7 (1 C), 114.6 (1 C), 117.3 (1 C), 119.4 (1 C), 121.2 (1 C), 127.6 (1 C), 130.0 (1 C), 132.0 (1 C), 132.3 (1 C), 132.4 (1 C), 134.0 (1 C), 136.5 (1 C), 137.8 (1 C), 143.3 (1 C), 146.5 (1 C), 149.3 (1 C), 158.7 (1 C), 158.9 (1 C), 159.9 (0.5 C), 161.6 (0.5 C), 163.9 (1 C), 167.0 (1 C), 167.5 (1 C), 169.1 (1 C), 170.3 (2 C),

173.0 (1 C).

***N*-(2-((2*S*,4*S*)-2-(((*S*)-1-(2-chloro-4-methoxyphenyl)ethyl)carbamoyl)-4-(2-((2-(2-(2-((2-(2,6-dioxopiperidin-3-yl)-1,3-dioxoisoindolin-4-yl)amino)ethoxy)ethoxy)ethyl)amino)-2-oxoethoxy)pyrrolidin-1-yl)-2-oxoethyl)-6-fluoroquinoline-2-carboxamide (12)** 2-(2,6-Dioxopiperidin-3-yl)-4-fluoroisoindoline-1,3-dione (100 mg, 0.362 mmol), *tert*-butyl (2-(2-(2-aminoethoxy)ethoxy)ethyl)carbamate (99 mg, 0.398 mmol) and DIPEA (252 μL) in DMF (1 mL) gave *tert*-butyl (2-(2-(2-((2-(2,6-dioxopiperidin-3-yl)-1,3-dioxoisoindolin-4-yl)amino)ethoxy)ethoxy)ethyl)carbamate (151 mg, 83%). Then, the resulting deBoc product, compound **8** (146 mg, 0.249 mmol), DIPEA (133 μL), and HATU (114 mg, 0.299 mmol) in DMF (2 mL) gave compound **12** (159 mg, 66%). HRMS calculated for $C_{45}H_{46}ClFN_8O_{11}$, 995.31132 (M+Na$^+$), found 995.31413. ^{1}H NMR (600 MHz, DMSO-d_6) δ ppm 1.30 (d, *J*=6.97 Hz, 2 H), 1.39 (d, *J*=7.15 Hz, 1 H), 1.80–1.86 (m, 0.7 H), 1.95–1.98 (m, 0.3 H), 2.01–2.05 (m, 1 H), 2.29–2.34 (m, 0.7 H), 2.52–2.62 (m, 2.3 H), 2.85–2.90 (m, 1 H), 3.23–3.29 (m, 2 H), 3.45 (d, *J*=4.77 Hz, 4.3 H), 3.52–3.55 (m, 2 H), 3.55–3.58 (m, 2 H), 3.60–3.62 (m, 2 H), 3.68 (br. s., 0.7 H), 3.70–3.73 (m, 2.3 H), 3.75 (s, 0.7 H), 3.80 (d, *J*=11.19 Hz, 1 H), 3.85–3.87 (m, 0.3 H), 3.88–3.98 (m, 2 H), 4.11–4.23 (m, 2 H), 4.27–4.33 (m, 0.7 H), 4.41 (t, *J*=7.70 Hz, 0.7 H), 4.64–4.58 (m, 0.3 H), 5.05 (dd, *J*=12.75, 5.23 Hz, 1 H), 5.10 (quin, *J*=7.34 Hz, 0.8 H), 5.18–5.24 (m, 0.2 H), 6.55–6.62 (m, 1 H), 6.82–6.91 (m, 1 H), 6.92–6.97 (m, 1 H), 7.01 (d, *J*=6.97 Hz, 1 H), 7.09–7.16 (m, 1 H), 7.30 (d, *J*=8.62 Hz, 0.7 H), 7.35–7.40 (m, 0.3 H), 7.55 (t, *J*=7.79 Hz, 1 H), 7.66–7.70 (br.s., 0.3 H), 7.71–7.76 (m, 0.7 H), 7.77–7.85 (m, 1 H), 7.93 (dd, *J*=8.99, 2.93 Hz, 1 H), 8.17–8.24 (m, 2 H), 8.43 (d, *J*=7.34 Hz, 0.7 H), 8.55–8.60 (m, 1 H), 8.80–8.84 (m, 0.3 H), 8.93–9.00 (m, 1 H), 11.09 (s, 1 H). ^{13}C NMR (151 MHz, DMSO-d_6) δ ppm 21.3 (1 C), 22.1 (1 C), 31.0 (1 C), 34.7 (1 C), 38.1 (1 C), 41.6 (1 C), 44.9 (1 C), 48.5 (1 C), 51.3 (1 C), 55.5 (2 C), 58.6 (1 C), 67.9 (1 C), 68.8 (1 C), 68.9 (1 C), 69.5 (1 C), 69.7 (1 C), 78.0 (1 C), 109.2 (1 C), 110.7 (1 C), 111.3 (1 C), 113.4 (1 C), 114.3 (1 C), 117.4 (1 C), 119.3 (1 C), 121.0 (1 C), 127.4 (1 C), 129.8 (1 C), 131.8 (1 C), 132.1 (1 C), 133.9 (1 C), 136.2 (1 C), 137.7 (1 C), 143.2 (1 C), 146.4 (1 C), 149.2 (1 C), 158.5 (1 C), 158.7 (1 C), 159.8 (0.5 C), 161.4 (0.5 C), 163.6 (1 C), 166.8 (1 C), 167.3 (1 C), 168.9 (2 C), 170.0 (1 C), 170.1 (1 C), 172.8 (1 C).

***N*-(2-((2*S*,4*S*)-2-(((*S*)-1-(2-chloro-4-methoxyphenyl)ethyl)carbamoyl)-4-(2-((8-((2-(2,6-dioxopiperidin-3-yl)-1,3-dioxoisoindolin-4-yl)amino)octyl)amino)-2-oxoethoxy)pyrrolidin-1-yl)-2-oxoethyl)-6-fluoroquinoline-2-carboxamide (13)** 2-(2,6-Dioxopiperidin-3-yl)-4-fluoroisoindoline-1,3-dione (100 mg, 0.362 mmol), *tert*-butyl (8-aminooctyl)carbamate (97 mg, 0.398 mmol) and DIPEA (252 μL) in DMF (1 mL) gave *tert*-butyl (8-((2-(2,6-dioxopiperidin-3-yl)-1,3-dioxoisoindolin-4-yl)amino)octyl)carbamate (146 mg, 81%). Then, the resulting deBoc product, compound **8** (143 mg, 0.243 mmol), DIPEA (130 μL), and HATU (111 mg, 0.282 mmol) in DMF (2 mL) gave compound **13** (168 mg, 71%). HRMS calculated for $C_{49}H_{54}ClFN_8O_{10}$, 991.35272 ($M+Na^+$), found 991.35337. ^1H NMR (600 MHz, DMSO-d_6) δ ppm 1.25–1.34 (m, 10 H), 1.36–1.45 (m, 3 H), 1.49–1.59 (m, 2 H), 1.81–1.86 (m, 0.7 H), 1.99–2.05 (m, 1.3 H), 2.33 (t, *J*=9.72 Hz, 0.7 H), 2.51–2.63 (m, 2.3 H), 2.84–2.92 (m, 1 H), 3.04–3.11 (m, 2 H), 3.21–3.27 (m, 2 H), 3.44–3.46 (m, 0.3 H), 3.69 (d, *J*=11.37 Hz, 0.7 H), 3.70–3.72 (m, 2.3 H), 3.73–3.75 (m, 0.7 H), 3.81 (d, *J*=10.45 Hz, 1 H), 3.84–3.86 (m, 0.3 H), 3.87–3.94 (m, 2 H), 4.12–4.23 (m, 2 H), 4.31 (dd, *J*=17.24, 5.32 Hz, 0.7 H), 4.42 (t, *J*=7.79 Hz, 0.7 H), 4.61–4.66 (m, 0.3 H), 5.05 (dd, *J*=12.84, 5.32 Hz, 1 H), 5.10 (quin, *J*=7.15 Hz, 0.7 H), 5.18–5.25 (m, 0.3 H), 6.49 (t, *J*=5.41 Hz, 1 H), 6.84 (dd, *J*=8.62, 2.38 Hz, 0.7 H), 6.91–6.95 (m, 0.3 H), 6.95 (d, *J*=2.57 Hz, 0.7 H), 6.97–7.03 (m, 1.3 H), 7.05 (d, *J*=8.62 Hz, 1 H), 7.30 (d, *J*=8.80 Hz, 0.7 H), 7.36–7.38 (m, 0.3 H), 7.56 (t, *J*=7.79 Hz, 1 H), 7.65–7.69 (m, 0.3 H), 7.73 (t, *J*=5.69 Hz, 0.7 H), 7.77–7.84 (m, 1 H), 7.89–7.96 (m, 1 H), 8.16–8.25 (m, 2 H), 8.43 (d, *J*=7.70 Hz, 0.7 H), 8.57 (d, *J*=8.44 Hz, 1 H), 8.79–8.84 (m, 0.3 H), 8.97 (t, *J*=5.04 Hz, 1 H), 11.10 (s, 1 H). ^{13}C NMR (151 MHz, DMSO-d_6) δ ppm 21.3 (1 C), 22.2 (1 C), 26.3 (1 C), 26.4 (1 C), 28.7 (1 C), 28.7 (2 C), 29.2 (1 C), 31.0 (1 C), 34.7 (1 C), 38.2 (1 C), 41.8 (1 C), 44.9 (1 C), 48.5 (1 C), 51.3 (1 C), 55.5 (2 C), 58.6 (1 C), 68.0 (1 C), 78.0 (1 C), 109.0 (1 C), 110.4 (1 C), 111.3 (1 C), 113.4 (1 C), 114.3 (1 C), 117.2 (1 C), 119.3 (1 C), 121.0 (1 C), 127.4 (1 C), 129.9 (1 C), 131.8 (1 C), 132.2 (1 C), 133.9 (1 C), 136.3 (1 C), 137.6 (1 C), 143.2 (1 C), 146.4 (1 C), 149.2 (1 C), 158.5 (1 C), 158.8 (1 C), 159.8 (0.5 C), 161.4 (0.5 C), 163.7 (1 C), 166.8 (1 C), 167.3 (1 C), 168.7 (1 C), 169.0 (1 C), 170.0 (1 C), 170.1 (1 C), 172.8 (1 C).

***N*-(2-((2*S*,4*S*)-2-(((*S*)-1-(2-chloro-4-methoxyphenyl)ethyl)carbamoyl)-4-((14-((2-(2,6-dioxopiperidin-3-yl)-1,3-dioxoisoindolin-4-yl)amino)-2-oxo-6,9,12-trioxa-3-azatetradecyl)oxy)pyrrolidin-1-yl)-2-oxoethyl)-6-fluoroquinoline-2-carboxamide (14)** 2-(2,6-Dioxopiperidin-3-yl)-4-fluoroisoindoline-1,3-dione (100 mg, 0.362 mmol), *tert*-butyl (2-(2-(2-(2-aminoethoxy)ethoxy)ethoxy)ethyl)carbamate (116 mg, 0.398 mmol) and DIPEA (252 μL) in DMF (1 mL) gave *tert*-butyl (2-(2-(2-(2-((2-(2,6-dioxopiperidin-3-yl)-1,3-dioxoisoindolin-4-yl)amino)ethoxy)ethoxy)ethoxy)ethyl)carbamate (151 mg, 76%). Then, the resulting deBoc product, compound **8** (135 mg, 0.230 mmol), DIPEA (123 μL), and HATU (105 mg, 0.276 mmol) in DMF (2 mL) gave compound **14** (178 mg, 76%). HRMS calculated for $C_{49}H_{54}ClFN_8O_{13}$, 1039.33752 (M+Na$^+$), found 1039.33940. ^{1}H NMR (600 MHz, DMSO-d_6) δ ppm 1.30 (d, *J*=7.15 Hz, 2.1 H), 1.37–1.40 (m, 0.9 H), 1.83 (ddd, *J*=13.20, 7.70, 4.95 Hz, 0.7 H), 1.96–1.99 (m, 0.3 H), 2.00–2.05 (m, 1 H), 2.30–2.35 (m, 0.7 H), 2.48–2.50 (m, 0.3 H), 2.51–2.62 (m, 2 H), 2.84–2.93 (m, 1 H), 3.22–3.28 (m, 2 H), 3.39–3.41 (m, 0.3 H), 3.41–3.46 (m, 4 H), 3.47–3.53 (m, 6 H), 3.53–3.56 (m, 2 H), 3.58–3.62 (m, 2 H), 3.68 (d, *J*=5.14 Hz, 0.7 H), 3.70–3.73 (m, 2.3 H), 3.75 (s, 0.7 H), 3.78–3.83 (m, 1 H), 3.84–3.87 (m, 0.3 H), 3.88–3.99 (m, 2 H), 4.11–4.24 (m, 2 H), 4.28–4.34 (m, 0.7 H), 4.41 (t, *J*=7.79 Hz, 0.7 H), 4.60–4.63 (m, 0.3 H), 5.05 (dd, *J*=12.75, 5.41 Hz, 1 H), 5.10 (quin, *J*=7.20 Hz, 0.7 H), 5.17–5.24 (m, 0.3 H), 6.56–6.61 (m, 1 H), 6.85 (dd, *J*=8.71, 2.48 Hz, 0.7 H), 6.88–6.91 (m, 0.3 H), 6.92–6.97 (m, 1 H), 6.99–7.04 (m, 1 H), 7.09–7.14 (m, 1 H), 7.30 (d, *J*=8.62 Hz, 0.7 H), 7.37–7.39 (m, 0.3 H), 7.56 (t, *J*=7.79 Hz, 1 H), 7.68–7.72 (m, 0.3 H), 7.75 (t, *J*=5.59 Hz, 0.7 H), 7.78–7.84 (m, 1 H), 7.90 - 7.95 (m, 1 H), 8.17–8.24 (m, 2 H), 8.43 (d, *J*=7.52 Hz, 0.7 H), 8.55–8.60 (m, 1 H), 8.80 - 8.85 (m, 0.3 H), 8.94–8.99 (m, 1 H), 11.09 (s, 1 H). ^{13}C NMR (151 MHz, DMSO-d_6) δ ppm 21.3 (1 C), 22.1 (1 C), 31.0 (1 C), 34.7 (1 C), 38.1 (1 C), 41.7 (1 C), 44.9 (1 C), 48.5 (1 C), 51.3 (1 C), 55.5 (2 C), 58.6(1 C), 67.9 (1 C), 68.9 (2 C), 69.6 (1 C), 69.8 (3 C), 78.0 (1 C), 109.2 (1 C), 110.6 (1 C), 111.3 (1 C), 113.4 (1 C), 114.3 (1 C), 117.4 (1 C), 119.3 (1 C), 121.0 (1 C), 127.4 (1 C), 129.9 (1 C), 131.8 (1 C), 132.2 (1 C), 133.9 (1 C), 136.2 (1 C), 137.7 (1 C), 143.2 (1 C), 146.4 (1 C), 149.2 (1 C), 158.5 (1 C), 158.7 (1 C), 159.8 (0.5 C), 161.4 (0.5 C), 163.6 (1 C), 166.8 (1 C), 167.3 (1 C), 168.9 (1 C), 169.0 (1 C), 170.0 (1 C), 170.1 (1 C), 172.8 (1 C).

***N*-(2-((2*S*,4*S*)-2-(((*S*)-1-(2-chloro-4-methoxyphenyl)ethyl)carbamoyl)-4-(2-((3-((1-**

(4-hydroxy-2-((4-(4-methylthiazol-5-yl)benzyl)carbamoyl)pyrrolidin-1-yl)-3,3-dimethyl-1-oxobutan-2-yl)amino)-3-oxopropyl)amino)-2-oxoethoxy)pyrrolidin-1-yl)-2-oxoethyl)-6-fluoroquinoline-2-carboxamide (15) To a solution of 1-(2-amino-3,3-dimethylbutanoyl)-4-hydroxy-*N*-(4-(4-methylthiazol-5-yl)benzyl)pyrrolidine-2-carboxamide (100 mg, 0.232 mmol), 3-((*tert*-butoxycarbonyl)amino)propanoic acid (44 mg, 0.232 mmol) and DIPEA (162 μL) in DMF (2 mL) was added HATU (115 mg, 0.302 mmol). The reaction mixture was stirred at room temperature for 4 h. The mixture was washed with sat. NaCl, extracted with ethyl acetate, dried over Na_2SO_4, concentrated and purified with flash column to give *tert*-butyl (3-((1-(4-hydroxy-2-((4-(4-methylthiazol-5-yl)benzyl)carbamoyl)pyrrolidin-1-yl)-3,3-dimethyl-1-oxobutan-2-yl)amino)-3-oxopropyl)carbamate (121 mg, 87%). The resulting residue was dissolved in CH_2Cl_2 (1 mL), followed by addition of TFA (2 mL). The mixture was stirred at room temperature for 3 h and concentrated *in vacuo*. The concentrated residue was redissolved in DMF (2 mL). Then, compound **8** (106 mg, 0.181 mmol), DIPEA (100 μL), and HATU (83 mg, 0.218 mmol) were added, and the reaction was stirred at room temperature for 3 h. The mixture was washed with sat. NaCl, extracted with ethyl acetate, dried over Na_2SO_4. The concentrated residue was purified with flash column to give the title compound (133 mg, 69%). HRMS calculated for $C_{53}H_{61}ClFN_9O_{10}S$, 1092.38272 (M+$Na^+$), found 1092.38352. ^1H NMR (600 MHz, DMSO-d_6) δ ppm 0.93 (s, 9 H), 1.31 (br. s., 2 H), 1.38–1.40 (m, 1 H), 1.80–1.86 (m, 0.7 H), 1.88 (ddd, *J*=12.88, 8.67, 4.68 Hz, 1 H), 1.96–2.00 (m, 0.3 H), 2.01–2.06 (m, 1 H), 2.30–2.35 (m, 0.8 H), 2.36–2.40 (m, 1 H), 2.43 (s, 3 H), 2.44–2.48 (m, 1 H), 2.53 (br. s., 0.2 H), 3.28–3.34 (m, 2 H), 3.43–3.46 (m, 1.3 H), 3.59–3.63 (m, 1 H), 3.67–3.70 (m, 0.7 H), 3.71 (s, 2.1 H), 3.74 (s, 0.9 H), 3.78–3.82 (m, 1 H), 3.83–3.86 (m, 0.3 H), 3.86–3.95 (m, 2 H), 4.10–4.26 (m, 3 H), 4.30 (d, *J*=4.95 Hz, 0.7 H), 4.32–4.35 (m, 1 H), 4.39–4.45 (m, 2.7 H), 4.52–4.58 (m, 1 H), 4.62–4.65 (m, 0.3 H), 5.05–5.17 (m, 1.7 H), 5.20–5.22 (m, 0.3 H), 6.84 (dd, *J*=8.62, 2.20 Hz, 0.7 H), 6.92–6.97 (m, 1 H), 6.99–7.00 (m, 0.3 H), 7.31 (d, *J*=8.62 Hz, 0.7 H), 7.35–7.39 (m, 2.3 H), 7.39–7.42 (m, 2 H), 7.67–7.69 (m, 0.3 H), 7.75 (t, *J*=5.50 Hz, 0.7 H), 7.79–7.84 (m, 1 H), 7.93 (d, *J*=9.35 Hz, 1 H), 7.99–8.03 (m, 1 H), 8.18–8.24 (m, 2 H), 8.44 (d, *J*=7.52 Hz, 0.7 H), 8.54–8.60 (m, 2 H), 8.83–8.84 (m, 0.3 H), 8.95–9.00 (m, 2 H). ^{13}C NMR (151 MHz, DMSO-d_6) δ ppm 15.9 (1 C), 21.3 (1 C), 26.3 (3 C), 34.7 (1 C), 35.1 (1 C), 35.3 (1 C), 37.9 (1 C), 40.1 (1 C), 41.6 (1 C), 44.9 (1 C), 51.3 (1 C), 55.5 (1 C), 55.5 (1 C), 56.4 (2 C), 58.6 (1 C), 58.7 (1 C), 67.9 (1

C), 68.9 (1 C), 78.0 (1 C), 111.2 (1 C), 113.4 (1 C), 114.3 (1 C), 119.3 (1 C), 121.0 (1 C), 127.4 (2 C), 128.6 (2 C), 129.6 (1 C), 129.9 (1 C), 131.1 (1 C), 131.8 (1 C), 132.2 (1 C), 133.9 (1 C), 137.6 (1 C), 139.5 (1 C), 143.2 (1 C), 147.7 (1 C), 149.2 (1 C), 151.4 (1 C), 158.5 (1 C), 158.7 (1 C), 159.8 (0.5 C), 161.4 (0.5 C), 163.6 (1 C), 166.8 (1 C), 168.7 (1 C), 169.5 (1 C), 170.0 (1 C), 170.4 (1 C), 171.9 (1 C).

Compound s16–22 was synthesized following the same procedure for compound 15

***N*-(2-((2*S*,4*S*)-2-(((*S*)-1-(2-chloro-4-methoxyphenyl)ethyl)carbamoyl)-4-(2-((5-((1-(4-hydroxy-2-((4-(4-methylthiazol-5-yl)benzyl)carbamoyl)pyrrolidin-1-yl)-3,3-dimethyl-1-oxobutan-2-yl)amino)-5-oxopentyl)amino)-2-oxoethoxy)pyrrolidin-1-yl)-2-oxoethyl)-6-fluoroquinoline-2-carboxamide (16)** 1-(2-Amino-3,3-dimethylbutanoyl)-4-hydroxy-*N*-(4-(4-methylthiazol-5-yl)benzyl)pyrrolidine-2-carboxamide (100 mg, 0.232 mmol), 5-((*tert*-butoxycarbonyl)amino)pentanoic acid (50 mg, 0.232 mmol), DIPEA (162 μL) and HATU (115 mg, 0.302 mmol) in DMF (1 mL) gave *tert*-butyl (5-((1-(4-hydroxy-2-((4-(4-methylthiazol-5-yl)benzyl)carbamoyl)pyrrolidin-1-yl)-3,3-dimethyl-1-oxobutan-2-yl)amino)-5-oxopentyl)carbamate (112 mg, 77%). Then, the resulting deBoc product, compound **8** (105 mg, 0.179 mmol), DIPEA (99 μL), and HATU (82 mg, 0.216 mmol) in DMF (2 mL) gave compound **16** (134 mg, 68%). HRMS calculated for $C_{55}H_{65}ClFN_9O_{10}S$, 1120.41402 (M+Na$^+$), found 1120.41499. ^{1}H NMR (600 MHz, DMSO-d_6) δ ppm 0.92 (s, 9 H), 1.31 (br. s., 2 H), 1.38–1.39 (m, 1 H), 1.39–1.45 (m, 2 H), 1.45–1.51 (m, 2 H), 1.80–1.86 (m, 0.7 H), 1.89 (ddd, *J*=12.75, 8.44, 4.49 Hz, 1 H), 1.96–2.00 (m, 0.3 H), 2.00–2.06 (m, 1 H), 2.09–2.19 (m, 1 H), 2.25 (dd, *J*=14.40, 7.61 Hz, 1 H), 2.32–2.37 (m, 0.7 H), 2.41–2.46 (m, 3 H), 2.53–2.55 (m, 0.3 H), 3.09 (d, *J*=6.97 Hz, 2 H), 3.41 (br. s., 1 H), 3.46–3.47 (m, 0.3 H), 3.64–3.66 (m, 1 H), 3.67–3.70 (m, 0.7 H), 3.70–3.72 (m, 2.3 H), 3.74 (s, 0.7 H), 3.80 (d, *J*=12.84 Hz, 1 H), 3.80–3.85 (m, 0.3 H), 3.89–3.94 (m, 2 H), 4.11–4.24 (m, 3 H), 4.30 (d, *J*=5.13 Hz, 0.7 H), 4.32–4.36 (m, 1 H), 4.39–4.44 (m, 2.7 H), 4.51–4.55 (m, 1 H), 4.62–4.64 (m, 0.3 H), 5.08–5.13 (m, 0.7 H), 5.15 (d, *J*=3.12 Hz, 1 H), 5.20–5.23 (m, 0.3 H), 6.84 (dd, *J*=8.80, 2.57 Hz, 0.7 H), 6.92–6.96 (m, 1 H), 6.99–7.00 (m, 0.3 H), 7.31 (d, *J*=8.62 Hz, 0.7 H), 7.36–7.39 (m, 2.3 H), 7.40–7.43 (m, 2 H), 7.74–7.76 (m, 0.3 H), 7.83 (m, 1.7 H), 7.86 (d, *J*=9.17 Hz, 1 H), 7.93

(dd, *J*=9.26, 2.66 Hz, 1 H), 8.18–8.24 (m, 2 H), 8.47 (d, *J*=7.52 Hz, 0.7 H), 8.56–8.60 (m, 2 H), 8.88–8.89 (m, 0.3 H), 8.95–8.99 (m, 2 H). ^{13}C NMR (151 MHz, DMSO-d_6) δ ppm 15.9 (1 C), 21.3 (1 C), 22.9 (1 C), 26.4 (3 C), 29.0 (1 C), 34.6 (1 C), 34.7 (1 C), 35.2 (1 C), 38.0 (2 C), 41.6 (1 C), 44.9 (1 C), 51.3 (1 C), 55.5 (1 C), 55.6 (1 C), 56.3 (1 C), 56.4 (1 C), 58.6 (1 C), 58.7 (1 C), 67.9 (1 C), 68.9 (1 C), 78.0 (1 C), 111.2 (1 C), 113.5 (1 C), 114.3 (1 C), 119.3 (1 C), 121.1 (1 C), 127.4 (2 C), 128.6 (2 C), 129.6 (1 C), 129.9 (1 C), 131.2 (1 C), 131.8 (1 C), 132.3 (1 C), 133.9 (1 C), 137.7 (1 C), 139.5 (1 C), 143.2 (1 C), 147.7 (1 C), 149.2 (1 C), 151.5 (1 C), 158.5 (1 C), 158.8 (1 C), 159.8 (0.5 C), 161.4 (0.5 C), 163.7 (1 C), 166.8 (1 C), 168.7 (1 C), 169.7 (1 C), 170.1 (1 C), 172.0 (1 C), 172.0 (1 C).

***N*-(2-((2*S*,4*S*)-2-(((*S*)-1-(2-chloro-4-methoxyphenyl)ethyl)carbamoyl)-4-(2-((2-(3-((1-(4-hydroxy-2-((4-(4-methylthiazol-5-yl)benzyl)carbamoyl)pyrrolidin-1-yl)-3,3-dimethyl-1-oxobutan-2-yl)amino)-3-oxopropoxy)ethyl)amino)-2-oxoethoxy)pyrrolidin-1-yl)-2-oxoethyl)-6-fluoroquinoline-2-carboxamide (17)** 1-(2-Amino-3,3-dimethylbutanoyl)-4-hydroxy-*N*-(4-(4-methylthiazol-5-yl)benzyl)pyrrolidine-2-carboxamide (100 mg, 0.232 mmol), 3-(2-((*tert*-butoxycarbonyl)amino)ethoxy)propanoic acid (54 mg, 0.232 mmol), DIPEA (162 μL) and HATU (115 mg, 0.302 mmol) in DMF (1 mL) gave *tert*-butyl (2-(3-((1-(4-hydroxy-2-((4-(4-methylthiazol-5-yl)benzyl)carbamoyl)pyrrolidin-1-yl)-3,3-dimethyl-1-oxobutan-2-yl)amino)-3-oxopropoxy)ethyl)carbamate (118 mg, 79%). Then, the resulting deBoc product, compound **8** (107 mg, 0.183 mmol), DIPEA (101 μL), and HATU (83 mg, 0.220 mmol) in DMF (2 mL) gave compound **17** (128 mg, 63%). HRMS calculated for $C_{55}H_{65}ClFN_9O_{11}S$, 1136.40892 (M+Na$^+$), found 1136.41185. ^{1}H NMR (600 MHz, DMSO-d_6) δ ppm 0.93 (br. s., 9 H), 1.30 (d, *J*=6.97 Hz, 2.1 H), 1.38 (br. s., 0.9 H), 1.84 (dd, *J*=12.84, 6.79 Hz, 0.7 H), 1.87–1.93 (m, 1 H), 1.98–2.00 (m, 0.3 H), 2.01–2.06 (m, 1 H), 2.30–2.34 (m, 0.7 H), 2.35–2.41 (m, 1 H), 2.43 (s, 3 H), 2.54 (d, *J*=7.70 Hz, 1.3 H), 3.25 (d, *J*=5.69 Hz, 2 H), 3.41 (d, *J*=5.87 Hz, 2 H), 3.45–3.48 (m, 0.3 H), 3.58–3.64 (m, 3 H), 3.67 (d, *J*=9.17 Hz, 1 H), 3.69–3.73 (m, 2.8 H), 3.75 (s, 0.9 H), 3.77–3.82 (m, 1 H), 3.87–3.88 (m, 0.3 H), 3.91–3.96 (m, 2 H), 4.13–4.24 (m, 3 H), 4.29–4.32 (m, 0.7 H), 4.35 (br. s., 1 H), 4.40–4.45 (m, 2.7 H), 4.55 (d, *J*=9.17 Hz, 1 H), 4.60–4.62 (m, 0.3 H), 5.08–5.12 (m, 0.7 H), 5.12–5.15 (m, 1 H), 5.20–5.22 (m, 0.3 H), 6.85 (d, *J*=8.25 Hz, 0.7 H), 6.92–6.97 (m, 1 H), 7.00 (br.s., 0.3 H), 7.31 (d, *J*=8.62 Hz,

0.7 H), 7.37 (m, *J*=7.89 Hz, 2.3 H), 7.41 (m, *J*=7.70 Hz, 2 H), 7.71–7.73 (m, 0.3 H), 7.76 (t, *J*=5.32 Hz, 0.7 H), 7.78–7.84 (m, 1 H), 7.91–7.96 (m, 2 H), 8.18–8.25 (m, 2 H), 8.43 (d, *J*=7.34 Hz, 0.8 H), 8.53–8.60 (m, 2 H), 8.82–8.84 (m, 0.2 H), 8.97 (s, 2 H). ^{13}C NMR (151 MHz, DMSO-d_6) δ ppm 15.9 (1 C), 21.3 (1 C), 26.3 (3 C), 34.7 (1 C), 35.3 (1 C), 35.6 (1 C), 37.9 (1 C), 38.0 (1 C), 41.6 (1 C), 44.9 (1 C), 51.2 (1 C), 55.5 (1 C), 55.5 (1 C), 56.3 (1 C), 56.4 (1 C), 58.6 (1 C), 58.7 (1 C), 66.7 (1 C), 67.9 (1 C), 68.6 (1 C), 68.9 (1 C), 78.0 (1 C), 111.2 (1 C), 113.4 (1 C), 114.3 (1 C), 119.3 (1 C), 120.9 (1 C), 127.4 (2 C), 128.6 (2 C), 129.6 (1 C), 129.8 (1 C), 131.1 (1 C), 131.8 (1 C), 132.2 (1 C), 133.9 (1 C), 137.7 (1 C), 139.5 (1 C), 143.2 (1 C), 147.7 (1 C), 149.2 (1 C), 151.4 (1 C), 158.5 (1 C), 158.7 (1 C), 159.8 (0.5 C), 161.4 (0.5 C), 163.6 (1 C), 166.8 (1 C), 169.0 (1 C), 169.5 (1 C), 169.9 (1 C), 170.0 (1 C), 171.9 (1 C).

***N*-(2-((2*S*,4*S*)-2-(((*S*)-1-(2-chloro-4-methoxyphenyl)ethyl)carbamoyl)-4-(2-((7-((1-(4-hydroxy-2-((4-(4-methylthiazol-5-yl)benzyl)carbamoyl)pyrrolidin-1-yl)-3,3-dimethyl-1-oxobutan-2-yl)amino)-7-oxoheptyl)amino)-2-oxoethoxy)pyrrolidin-1-yl)-2-oxoethyl)-6-fluoroquinoline-2-carboxamide (18)** 1-(2-Amino-3,3-dimethylbutanoyl)-4-hydroxy-*N*-(4-(4-methylthiazol-5-yl)benzyl)pyrrolidine-2-carboxamide (100 mg, 0.232 mmol), 7-((*tert*-butoxycarbonyl)amino)heptanoic acid (57 mg, 0.232 mmol), DIPEA (162 μL) and HATU (115 mg, 0.302 mmol) in DMF (1 mL) gave *tert*-butyl (7-((1-(4-hydroxy-2-((4-(4-methylthiazol-5-yl)benzyl)carbamoyl)pyrrolidin-1-yl)-3,3-dimethyl-1-oxobutan-2-yl)amino)-7-oxoheptyl)carbamate (120 mg, 79%). Then, the resulting deBoc product, compound **8** (107 mg, 0.183 mmol), DIPEA (101 μL), and HATU (83 mg, 0.220 mmol) in DMF (2 mL) gave compound **18** (139 mg, 67%). HRMS calculated for $C_{57}H_{69}ClFN_9O_{10}S$, 1148.44532 (M+Na$^+$), found 1148.44838. ^{1}H NMR (600 MHz, DMSO-d_6) δ ppm 0.92 (s, 9 H), 1.27–1.32 (m, 4 H), 1.37–1.42 (m, 4 H), 1.42–1.53 (m, 3 H), 1.82 (ddd, *J*=13.16, 7.75, 4.95 Hz, 0.7 H), 1.89 (ddd, *J*=12.61, 8.30, 4.77 Hz, 1 H), 1.97–1.99 (m, 0.3 H), 2.00–2.06 (m, 1 H), 2.07–2.14 (m, 1 H), 2.24 (dt, *J*=14.21, 7.38 Hz, 1 H), 2.31–2.36 (m, 0.7 H), 2.43–2.44 (m, 3 H), 2.52–2.55 (m, 0.3 H), 3.03–3.10 (m, 2 H), 3.40 (t, *J*=4.22 Hz, 1 H), 3.43–3.45 (m, 0.3 H), 3.65 (d, *J*=7.89 Hz, 1 H), 3.68 (t, *J*=4.86 Hz, 0.7 H), 3.70–3.72 (m, 2.3 H), 3.75 (s, 0.7 H), 3.77–3.81 (m, 1 H), 3.82–3.84 (m, 0.3 H), 3.84–3.94 (m, 2 H), 4.11–4.24 (m, 3 H), 4.30 (d, *J*=5.32 Hz, 0.7 H), 4.33–4.36 (m, 1 H), 4.40–4.44 (m, 2.7 H), 4.54 (d, *J*=9.35 Hz, 1 H), 4.61–4.63 (m, 0.3 H), 5.10 (t, *J*=7.15

Hz, 0.7 H), 5.14 (d, *J*=3.12 Hz, 1 H), 5.19–5.22 (m, 0.3 H), 6.85 (dd, *J*=8.62, 2.57 Hz, 0.7 H), 6.92–6.96 (m, 1 H), 6.99–7.00 (m, 0.3 H), 7.31 (d, *J*=8.80 Hz, 0.7 H), 7.36–7.39 (m, 2.3 H), 7.40–7.42 (m, 2 H), 7.68–7.72 (m, 0.3 H), 7.75 (t, *J*=5.78 Hz, 0.7 H), 7.78–7.82 (m, 1 H), 7.82–7.87 (m, 1 H), 7.93 (dd, *J*=9.26, 2.66 Hz, 1 H), 8.18–8.25 (m, 2 H), 8.43–8.47 (m, 0.7 H), 8.55–8.60 (m, 2 H), 8.84–8.86 (m, 0.3 H), 8.95–9.00 (m, 2 H). ^{13}C NMR (151 MHz, DMSO-d_6) δ ppm 16.0 (1 C), 21.3 (1 C), 25.4 (1 C), 26.2 (1 C), 26.4 (3 C), 28.4 (1 C), 29.2 (1 C), 34.7 (1 C), 34.8 (1 C), 35.2 (1 C), 38.0 (1 C), 38.2 (1 C), 41.7 (1 C), 44.9 (1 C), 51.3 (1 C), 55.5 (1 C), 55.6 (1 C), 56.3 (1 C), 56.4 (1 C), 58.6 (1 C), 58.7 (1 C), 67.9 (1 C), 68.9 (1 C), 78.0 (1 C), 111.2 (1 C), 113.5 (1 C), 114.3 (1 C), 119.3 (1 C), 121.1 (1 C), 127.4 (2 C), 128.6 (2 C), 129.6 (1 C), 129.9 (1 C), 131.2 (1 C), 131.8 (1 C), 132.3 (1 C), 133.9 (1 C), 137.7 (1 C), 139.5 (1 C), 143.2 (1 C), 147.7 (1 C), 149.2 (1 C), 151.5 (1 C), 158.5 (1 C), 158.8 (1 C), 159.8 (0.5 C), 161.4 (0.5 C), 163.7 (1 C), 166.8 (1 C), 168.7 (1 C), 169.7 (1 C), 170.1 (1 C), 172.0 (1 C), 172.1 (1 C).

***N*-(2-((2*S*,4*S*)-2-(((*S*)-1-(2-chloro-4-methoxyphenyl)ethyl)carbamoyl)-4-((14-(4-hydroxy-2-((4-(4-methylthiazol-5-yl)benzyl)carbamoyl)pyrrolidine-1-carbonyl)-15,15-dimethyl-2,12-dioxo-6,9-dioxa-3,13-diazahexadecyl)oxy)pyrrolidin-1-yl)-2-oxoethyl)-6-fluoroquinoline-2-carboxamide (19)** 1-(2-Amino-3,3-dimethylbutanoyl)-4-hydroxy-*N*-(4-(4-methylthiazol-5-yl)benzyl)pyrrolidine-2-carboxamide (100 mg, 0.232 mmol), 2,2-dimethyl-4-oxo-3,8,11-trioxa-5-azatetradecan-14-oic acid (64 mg, 0.232 mmol), DIPEA (162 μL) and HATU (115 mg, 0.302 mmol) in DMF (1 mL) gave *tert*-butyl (2-(2-(3-((1-(4-hydroxy-2-((4-(4-methylthiazol-5-yl)benzyl)carbamoyl)pyrrolidin-1-yl)-3,3-dimethyl-1-oxobutan-2-yl)amino)-3-oxopropoxy)ethoxy)ethyl)carbamate (117 mg, 73%). Then, the resulting deBoc product, compound **8** (99 mg, 0.170 mmol), DIPEA (94 μL), and HATU (78 mg, 0.204 mmol) in DMF (2 mL) gave compound **19** (138 mg, 70%). HRMS calculated for $C_{57}H_{69}ClFN_9O_{12}S$, 1180.43512 (M+Na$^+$), found 1180.43654. ^{1}H NMR (600 MHz, DMSO-d_6) δ ppm 0.92 (s, 9 H), 1.30 (d, *J*=7.34 Hz, 2.2 H), 1.39 (d, *J*=6.97 Hz, 0.8 H), 1.83 (ddd, *J*=13.20, 7.70, 4.95 Hz, 0.7 H), 1.89 (ddd, *J*=12.70, 8.48, 4.68 Hz, 1 H), 1.97–2.00 (m, 0.3 H), 2.01–2.06 (m, 1 H), 2.29–2.33 (m, 0.7 H), 2.34–2.40 (m, 1 H), 2.43 (s, 3 H), 2.51–2.56 (m, 1.3 H), 3.22–3.28 (m, 2 H), 3.39–3.43 (m, 2.3 H), 3.45–3.52 (m, 4 H), 3.57 (dt, *J*=6.56, 3.42 Hz, 1 H), 3.60–3.64 (m, 2 H), 3.64–3.69 (m, 1 H),

3.70 (br. s., 0.7 H), 3.71 (s, 2.1 H), 3.75 (s, 0.9 H), 3.81 (d, *J*=11.92 Hz, 1 H), 3.85–3.87 (m, 0.3 H), 3.89–3.96 (m, 2 H), 4.13–4.24 (m, 3 H), 4.31 (d, *J*=12.10 Hz, 0.7 H), 4.34 (br. s., 1 H), 4.39–4.45 (m, 2.7 H), 4.55 (d, *J*=9.35 Hz, 1 H), 4.61 4.63 (m, 0.3 H), 5.07–5.12 (m, 0.7 H), 5.14 (d, *J*=3.30 Hz, 1 H), 5.20–5.22 (m, 0.3 H), 6.85 (dd, *J*=8.71, 2.48 Hz, 0.7 H), 6.92–6.97 (m, 1 H), 6.99–7.00 (m, 0.3 H), 7.31 (d, *J*=8.62 Hz, 0.7 H), 7.37 (d, *J*=8.25 Hz, 2.3 H), 7.41 (d, *J*=8.25 Hz, 2 H), 7.70–7.73 (m, 0.3 H), 7.77 (t, *J*=5.69 Hz, 0.7 H), 7.81 (td, *J*=8.85, 3.03 Hz, 1 H), 7.91–7.95 (m, 2 H), 8.18–8.24 (m, 2 H), 8.45 (d, *J*=7.52 Hz, 0.7 H), 8.55–8.60 (m, 2 H), 8.83–8.86 (m, 0.3 H), 8.95–9.00 (m, 2 H). ^{13}C NMR (151 MHz, DMSO-d_6) δ ppm 15.9 (1 C), 21.3 (1 C), 26.3 (3 C), 34.7 (1 C), 35.4 (1 C), 35.6 (1 C), 38.0 (1 C), 38.1 (1 C), 41.6 (1 C), 44.9 (1 C), 51.3 (1 C), 55.5 (1 C), 55.6 (1 C), 56.3 (1 C), 56.4 (1 C), 58.6 (1 C), 58.7 (1 C), 66.9 (1 C), 67.9 (1 C), 68.9 (1 C), 68.9 (1 C), 69.4 (1 C), 69.5 (1 C), 78.0 (1 C), 111.2 (1 C), 113.5 (1 C), 114.3 (1 C), 119.3 (1 C), 121.1 (1 C), 127.4 (2 C), 128.6 (2 C), 129.6 (1 C), 129.8 (1 C), 131.2 (1 C), 131.8 (1 C), 132.3 (1 C), 133.9 (1 C), 137.7 (1 C), 139.5 (1 C), 143.2 (1 C), 147.7 (1 C), 149.2 (1 C), 151.5 (1 C), 158.5 (1 C), 158.8 (1 C), 159.8 (0.5 C), 161.4 (0.5 C), 163.7 (1 C), 166.8 (1 C), 169.0 (1 C), 169.5 (1 C), 170.0 (1 C), 170.0 (1 C), 171.9 (1 C).

***N*-(2-((2*S*,4*S*)-2-(((*S*)-1-(2-chloro-4-methoxyphenyl)ethyl)carbamoyl)-4-(2-((9-((1-(4-hydroxy-2-((4-(4-methylthiazol-5-yl)benzyl)carbamoyl)pyrrolidin-1-yl)-3,3-dimethyl-1-oxobutan-2-yl)amino)-9-oxononyl)amino)-2-oxoethoxy)pyrrolidin-1-yl)-2-oxoethyl)-6-fluoroquinoline-2-carboxamide (20)** 1-(2-Amino-3,3-dimethylbutanoyl)-4-hydroxy-*N*-(4-(4-methylthiazol-5-yl)benzyl)pyrrolidine-2-carboxamide (100 mg, 0.232 mmol), 9-((*tert*-butoxycarbonyl)amino)nonanoic acid (63 mg, 0.232 mmol), DIPEA (162 μL) and HATU (115 mg, 0.302 mmol) in DMF (1 mL) gave *tert*-butyl (9-((1-(4-hydroxy-2-((4-(4-methylthiazol-5-yl)benzyl)carbamoyl)pyrrolidin-1-yl)-3,3-dimethyl-1-oxobutan-2-yl)amino)-9-oxononyl)carbamate (122 mg, 77%). Then, the resulting deBoc product, compound **8** (104 mg, 0.178 mmol), DIPEA (98 μL), and HATU (81 mg, 0.214 mmol) in DMF (2 mL) gave compound **20** (130 mg, 63%). HRMS calculated for $C_{59}H_{73}ClFN_9O_{10}S$, 1176.47662 (M+Na$^+$), found 1176.47568. ^{1}H NMR (600 MHz, DMSO-d_6) δ ppm 0.93 (s, 9 H), 1.28–1.32 (m, 5 H), 1.33–1.36 (m, 2 H), 1.36–1.45 (m, 5 H), 1.47 (dd, *J*=13.39, 7.15 Hz, 2 H), 1.54 (d, *J*=6.97 Hz, 1 H), 1.80–1.86 (m, 0.7 H), 1.87–1.93 (m, 1 H),

1.96–1.98 (m, 0.3 H), 2.00–2.05 (m, 1 H), 2.07–2.13 (m, 1 H), 2.24 (dq, *J*=14.21, 7.00 Hz, 1 H), 2.30–2.36 (m, 0.7 H), 2.43–2.45 (m, 3 H), 2.54–2.55 (m, 0.3 H), 3.04–3.10 (m, 2 H), 3.42–3.47 (m, 1.3 H), 3.64–3.66 (m, 1 H), 3.68 (br. s., 0.7 H), 3.70–3.72 (m, 2.3 H), 3.75 (s, 0.7 H), 3.79–3.83 (m, 1.3 H), 3.83–3.94 (m, 2 H), 4.11–4.25 (m, 3 H), 4.29–4.33 (m, 0.7 H), 4.35 (br. s., 1 H), 4.40–4.45 (m, 2.7 H), 4.54 (d, *J*=9.35 Hz, 1 H), 4.63–4.65 (m, 0.3 H), 5.06–5.17 (m, 1.7 H), 5.20–5.22 (m, 0.3 H), 6.85 (dd, *J*=8.62, 2.38 Hz, 0.7 H), 6.91–6.97 (m, 1 H), 6.99–7.00 (m, 0.3 H), 7.31 (d, *J*=8.62 Hz, 0.7 H), 7.38 (d, *J*=8.07 Hz, 2.3 H), 7.41 (d, *J*=8.25 Hz, 2 H), 7.67–7.69 (m, 0.3 H), 7.75 (t, *J*=5.69 Hz, 0.7 H), 7.79–7.87 (m, 2 H), 7.91–7.96 (m, 1 H), 8.18–8.25 (m, 2 H), 8.45 (d, *J*=7.70 Hz, 0.7 H), 8.55–8.59 (m, 2 H), 8.83–8.85 (m, 0.3 H), 8.96–9.00 (m, 2 H). ^{13}C NMR (151 MHz, DMSO-d_6) δ ppm 15.9 (1 C), 21.3 (1 C), 25.4 (1 C), 26.4 (4 C), 28.7 (1 C), 29.3 (1 C), 29.8 (1 C), 30.4 (1 C), 34.7 (1 C), 34.9 (1 C), 35.2 (1 C), 38.0 (1 C), 38.2 (1 C), 41.6 (1 C), 44.9 (1 C), 51.3 (1 C), 55.5 (1 C), 55.6 (1 C), 56.3 (1 C), 56.4 (1 C), 58.6 (1 C), 58.7 (1 C), 67.9 (1 C), 68.9 (1 C), 78.0 (1 C), 111.2 (1 C), 113.4 (1 C), 114.3 (1 C), 119.3 (1 C), 121.1 (1 C), 127.4 (2 C), 128.6 (2 C), 129.6 (1 C), 129.9 (1 C), 131.2 (1 C), 131.8 (1 C), 132.3 (1 C), 133.9 (1 C), 137.7 (1 C), 139.5 (1 C), 143.2 (1 C), 147.7 (1 C), 149.2 (1 C), 151.5 (1 C), 158.5 (1 C), 158.8 (1 C), 159.8 (0.5 C), 161.4 (0.5 C), 163.7 (1 C), 166.8 (1 C), 168.7 (1 C), 169.7 (1 C), 170.1 (1 C), 172.0 (1 C), 172.1 (1 C).

***N*-(2-((2*S*,4*S*)-2-(((*S*)-1-(2-chloro-4-methoxyphenyl)ethyl)carbamoyl)-4-((17-(4-hydroxy-2-((4-(4-methylthiazol-5-yl)benzyl)carbamoyl)pyrrolidine-1-carbonyl)-18,18-dimethyl-2,15-dioxo-6,9,12-trioxa-3,16-diazanonadecyl)oxy)pyrrolidin-1-yl)-2-oxoethyl)-6-fluoroquinoline-2-carboxamide (21)** 1-(2-Amino-3,3-dimethylbutanoyl)-4-hydroxy-*N*-(4-(4-methylthiazol-5-yl)benzyl)pyrrolidine-2-carboxamide (100 mg, 0.232 mmol), 2,2-dimethyl-4-oxo-3,8,11,14-tetraoxa-5-azaheptadecan-17-oic acid (75 mg, 0.232 mmol), DIPEA (162 μL) and HATU (115 mg, 0.302 mmol) in DMF (1 mL) gave *tert*-butyl (14-(4-hydroxy-2-((4-(4-methylthiazol-5-yl)benzyl)carbamoyl)pyrrolidine-1-carbonyl)-15,15-dimethyl-12-oxo-3,6,9-trioxa-13-azahexadecyl)carbamate (121 mg, 71%). Then, the resulting deBoc product, compound **8** (97 mg, 0.165 mmol), DIPEA (91 μL), and HATU (75 mg, 0.198 mmol) in DMF (2 mL) gave compound **21** (127 mg, 64%). HRMS calculated for $C_{59}H_{73}ClFN_9O_{13}S$, 1224.46132 (M+Na$^+$), found 1224.46469. ^{1}H NMR (600 MHz, DMSO-d_6) δ ppm 0.92

(s, 9 H), 1.30 (d, *J*=7.34 Hz, 2.1 H), 1.38–1.39 (m, 0.9 H), 1.83 (ddd, *J*=13.25, 7.75, 5.04 Hz, 0.7 H), 1.90 (ddd, *J*=12.70, 8.48, 4.49 Hz, 1 H), 1.95–1.97 (m, 0.3 H), 2.00–2.06 (m, 1 H), 2.30–2.33 (m, 0.7 H), 2.33–2.38 (m, 1 H), 2.43 (s, 3 H), 2.51–2.57 (m, 1.3 H), 3.22–3.29 (m, 2 H), 3.41–3.44 (m, 2.3 H), 3.46–3.49 (m, 8 H), 3.54–3.60 (m, 2 H), 3.62 (d, *J*=11.37 Hz, 1 H), 3.65–3.69 (m, 1 H), 3.70 (br. s., 0.7 H), 3.71 (s, 1.8 H), 3.75 (d, *J*=3.48 Hz, 1.2 H), 3.81 (d, *J*=11.00 Hz, 1 H), 3.85–3.87 (m, 0.3 H), 3.88–3.99 (m, 2 H), 4.13–4.24 (m, 3 H), 4.29–4.33 (m, 0.7 H), 4.35 (br. s., 1 H), 4.40–4.45 (m, 2.7 H), 4.54 (d, *J*=9.35 Hz, 1 H), 4.60–4.63 (m, 0.3 H), 5.07–5.13 (m, 0.7 H), 5.14 (d, *J*=3.48 Hz, 1 H), 5.21–5.22 (m, 0.3 H), 6.85 (dd, *J*=8.71, 2.48 Hz, 0.6 H), 6.93–6.97 (m, 1.2 H), 7.00–7.01 (m, 0.2 H), 7.30 (d, *J*=8.62 Hz, 0.7 H), 7.36–7.39 (m, 2.3 H), 7.40–7.43 (m, 2 H), 7.72–7.76 (m, 0.3 H), 7.77 (t, *J*=5.78 Hz, 0.7 H), 7.79–7.84 (m, 1 H), 7.90–7.95 (m, 2 H), 8.17–8.25 (m, 2 H), 8.42–8.47 (m, 0.7 H), 8.55–8.60 (m, 2 H), 8.82–8.84 (m, 0.3 H), 8.95–9.01 (m, 2 H). ^{13}C NMR (151 MHz, DMSO-d_6) δ ppm 15.9 (1 C), 21.3 (1 C), 26.3 (3 C), 34.7 (1 C), 35.4 (1 C), 35.6 (1 C), 38.0 (1 C), 38.1 (1 C), 41.7 (1 C), 44.9 (1 C), 51.3 (1 C), 55.5 (1 C), 55.6 (1 C), 56.3 (1 C), 56.4 (1 C), 58.6 (1 C), 58.7 (1 C), 66.9 (1 C), 67.9 (1 C), 68.9 (1 C), 68.9 (1 C), 69.5 (1 C), 69.5 (1 C), 69.7 (1 C), 69.7 (1 C), 78.0 (1 C), 111.2 (1 C), 113.5 (1 C), 114.3 (1 C), 119.3 (1 C), 121.1 (1 C), 127.4 (2 C), 128.7 (2 C), 129.7 (1 C), 129.9 (1 C), 131.2 (1 C), 131.8 (1 C), 132.3 (1 C), 133.9 (1 C), 137.7 (1 C), 139.5 (1 C), 143.2 (1 C), 147.7 (1 C), 149.2 (1 C), 151.5 (1 C), 158.5 (1 C), 158.8 (1 C), 159.8 (0.5 C), 161.4 (0.5 C), 163.7 (1 C), 166.8 (1 C), 169.0 (1 C), 169.5 (1 C), 170.0 (1 C), 170.1 (1 C), 172.0 (1 C).

***N*-(2-((2*S*,4*S*)-2-(((*S*)-1-(2-chloro-4-methoxyphenyl)ethyl)carbamoyl)-4-((14-(4-hydroxy-2-((1-(4-(4-methylthiazol-5-yl)phenyl)ethyl)carbamoyl)pyrrolidine-1-carbonyl)-15,15-dimethyl-2,12-dioxo-6,9-dioxa-3,13-diazahexadecyl)oxy)pyrrolidin-1-yl)-2-oxoethyl)-6-fluoroquinoline-2-carboxamide (22)** 1-(2-Amino-3,3-dimethylbutanoyl)-4-hydroxy-*N*-(1-(4-(4-methylthiazol-5-yl)phenyl)ethyl)pyrrolidine-2-carboxamide (100 mg, 0.225 mmol), 2,2-dimethyl-4-oxo-3,8,11-trioxa-5-azatetradecan-14-oic acid (62 mg, 0.225 mmol), DIPEA (157 μL) and HATU (112 mg, 0.293 mmol) in DMF (1 mL) gave *tert*-butyl (2-(2-(3-((1-(4-hydroxy-2-((1-(4-(4-methylthiazol-5-yl)phenyl)ethyl)carbamoyl)pyrrolidin-1-yl)-3,3-dimethyl-1-oxobutan-2-yl)amino)-3-oxopropoxy)ethoxy)ethyl)carbamate (118 mg, 75%). Then, the resulting deBoc product,

compound **8** (98 mg, 0.168 mmol), DIPEA (93 μL), and HATU (77 mg, 0.202 mmol) in DMF (2 mL) gave compound **22** (133 mg, 68%). HRMS calculated for $C_{58}H_{71}ClFN_9O_{12}S$, 1194.45072 ($M+Na^+$), found 1194.45212. ^1H NMR (600 MHz, DMSO-d_6) δ ppm 0.92 (br. s., 9 H), 1.30 (d, *J*=7.52 Hz, 2.1s H), 1.33 (s, 0.9 H), 1.36 (d, *J*=6.60 Hz, 2.1 H), 1.39 (s, 0.9 H), 1.76–1.81 (m, 1 H), 1.81–1.87 (m, 0.7 H), 1.98–2.00 (m, 0.3 H), 2.00–2.04 (m, 1 H), 2.33 (br. s., 0.7 H), 2.34–2.39 (m, 1 H), 2.45 (s, 3 H), 2.51–2.57 (m, 1.3 H), 3.25–3.28 (m, 2 H), 3.40–3.41 (m, 0.3 H), 3.43 (t, *J*=6.05 Hz, 2 H), 3.47–3.50 (m, 4 H), 3.56 (br. s., 2 H), 3.63–3.65 (m, 2 H), 3.69 (d, *J*=3.67 Hz, 0.7 H), 3.71 (s, 2.2 H), 3.75 (s, 0.8 H), 3.82 (br. s., 1 H), 3.85–3.88 (m, 0.3 H), 3.89–3.99 (m, 2 H), 4.12–4.24 (m, 2 H), 4.27 (br. s., 1 H), 4.29–4.35 (m, 0.7 H), 4.42 (t, *J*=7.79 Hz, 1.7 H), 4.52 (d, *J*=9.17 Hz, 1 H), 4.61–4.63 (m, 0.3 H), 4.91 (quin, *J*=7.11 Hz, 1 H), 5.09–5.13 (m, 1.7 H), 5.20–5.22 (m, 0.3 H), 6.85 (dd, *J*=8.62, 2.20 Hz, 0.7 H), 6.92–6.97 (m, 1 H), 6.99–7.02 (m, 0.3 H), 7.31 (d, *J*=8.62 Hz, 0.7 H), 7.33–7.34 (m, 0.3 H), 7.36–7.38 (m, 2 H), 7.42 (d, *J*=7.52 Hz, 2 H), 7.71–7.73 (m, 0.3 H), 7.77 (t, *J*=5.50 Hz, 0.7 H), 7.81 (td, *J*=8.71, 2.57 Hz, 1 H), 7.86 (d, *J*=8.99 Hz, 1 H), 7.91–7.95 (m, 1 H), 8.22 (q, *J*=8.56 Hz, 2 H), 8.38 (d, *J*=7.70 Hz, 1 H), 8.45 (br. s., 0.7 H), 8.58 (d, *J*=8.44 Hz, 1 H), 8.83–8.85 (m, 0.3 H), 8.95–9.00 (m, 2 H). ^{13}C NMR (151 MHz, DMSO-d_6) δ ppm 16.0 (1 C), 21.3 (1 C), 22.4 (1 C), 26.4 (3 C), 34.7 (1 C), 35.3 (1 C), 35.6 (1 C), 37.7 (1 C), 38.1 (1 C), 41.8 (1 C), 46.3 (1 C), 51.3 (1 C), 55.5 (2 C), 56.3 (1 C), 56.4 (1 C), 58.5 (1 C), 58.6 (1 C), 66.9 (1 C), 67.9 (1 C), 68.7 (1 C), 68.9 (1 C), 69.4 (1 C), 69.5 (1 C), 78.0 (1 C), 111.2 (1 C), 113.4 (1 C), 114.3 (1 C), 119.3 (1 C), 121.0 (1 C), 126.4 (2 C), 128.8 (2 C), 129.7 (1 C), 129.9 (1 C), 131.1 (1 C), 131.8 (1 C), 132.2 (1 C), 133.9 (1 C), 137.7 (1 C), 143.2 (1 C), 144.7 (1 C), 147.7 (1 C), 149.2 (1 C), 151.5 (1 C), 158.5 (1 C), 158.7 (1 C), 159.8 (0.5 C), 161.4 (0.5 C), 163.6 (1 C), 166.8 (1 C), 169.0 (1 C), 169.4 (1 C), 169.9 (1 C), 170.0 (1 C), 170.6 (1 C).

Reference

1. Scott DE, Francis-Newton NJ, Marsh ME, Coyne AG, Fischer G, Moschetti T, et al. A small molecule inhibitor of the BRCA2-RAD51 interaction modulates RAD51 assembly and potentiates DNA damage-induced cell death. *Cell Chem Biol* 2021;**28**:835–47.e5.

4. Spectra of active compound G73

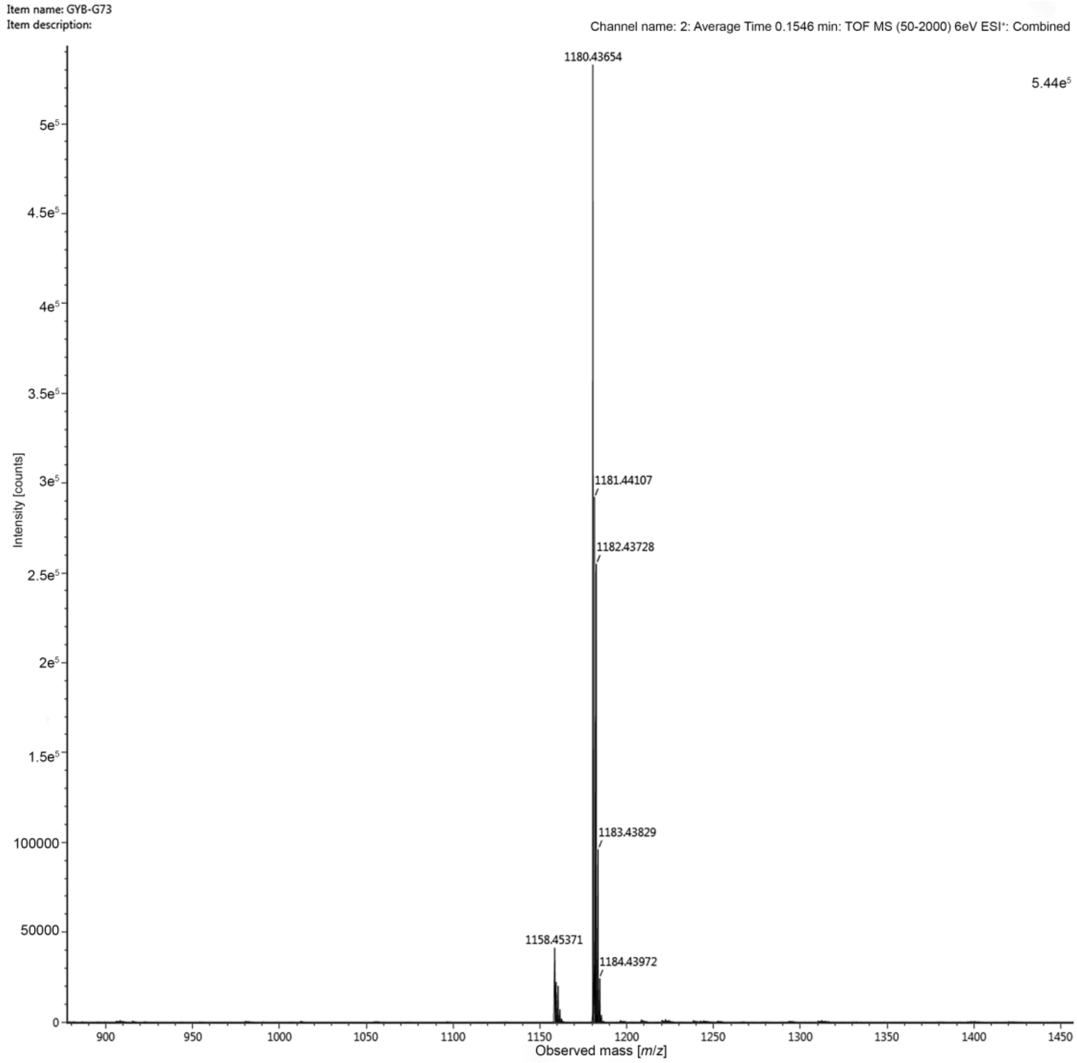

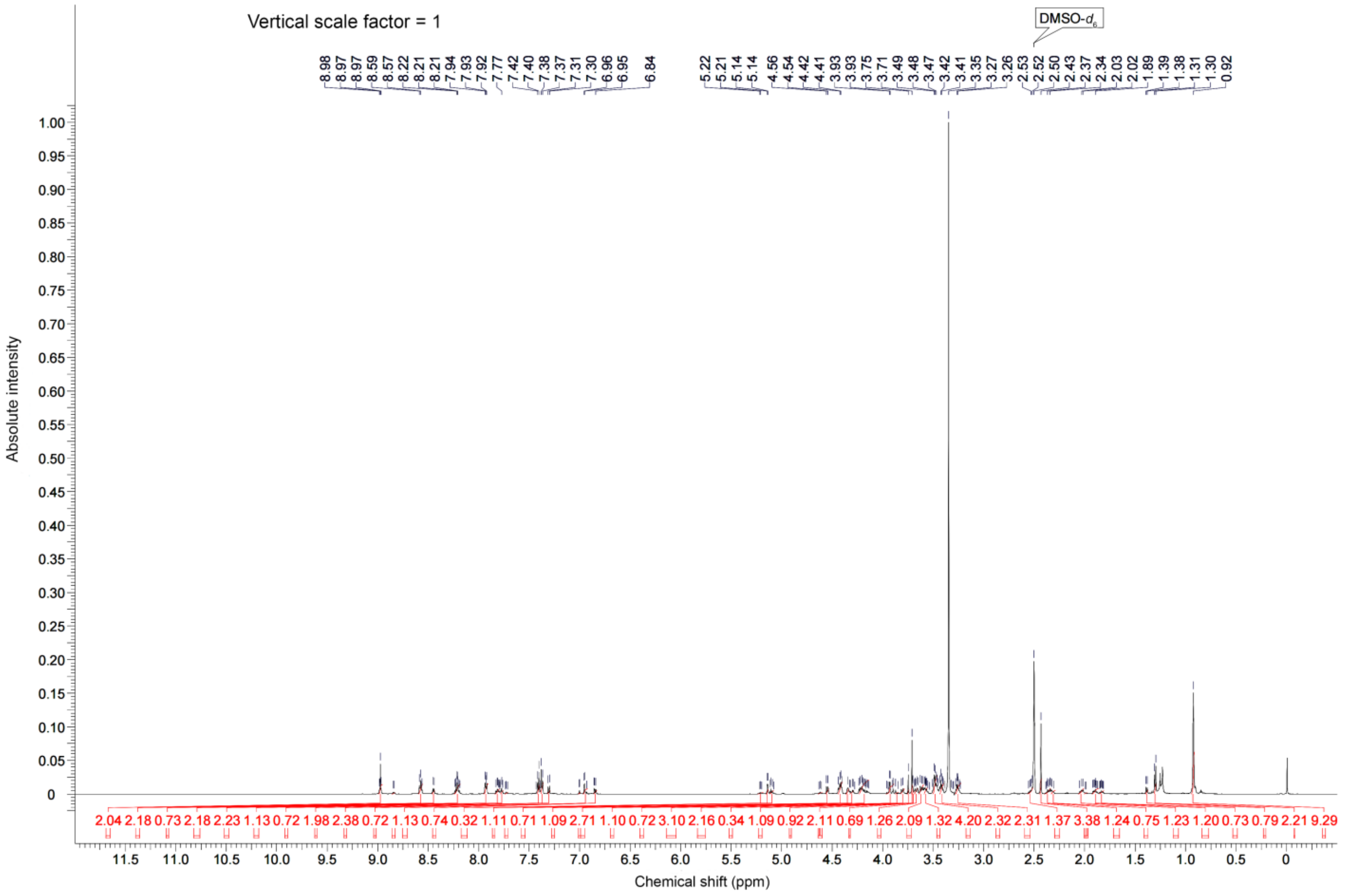

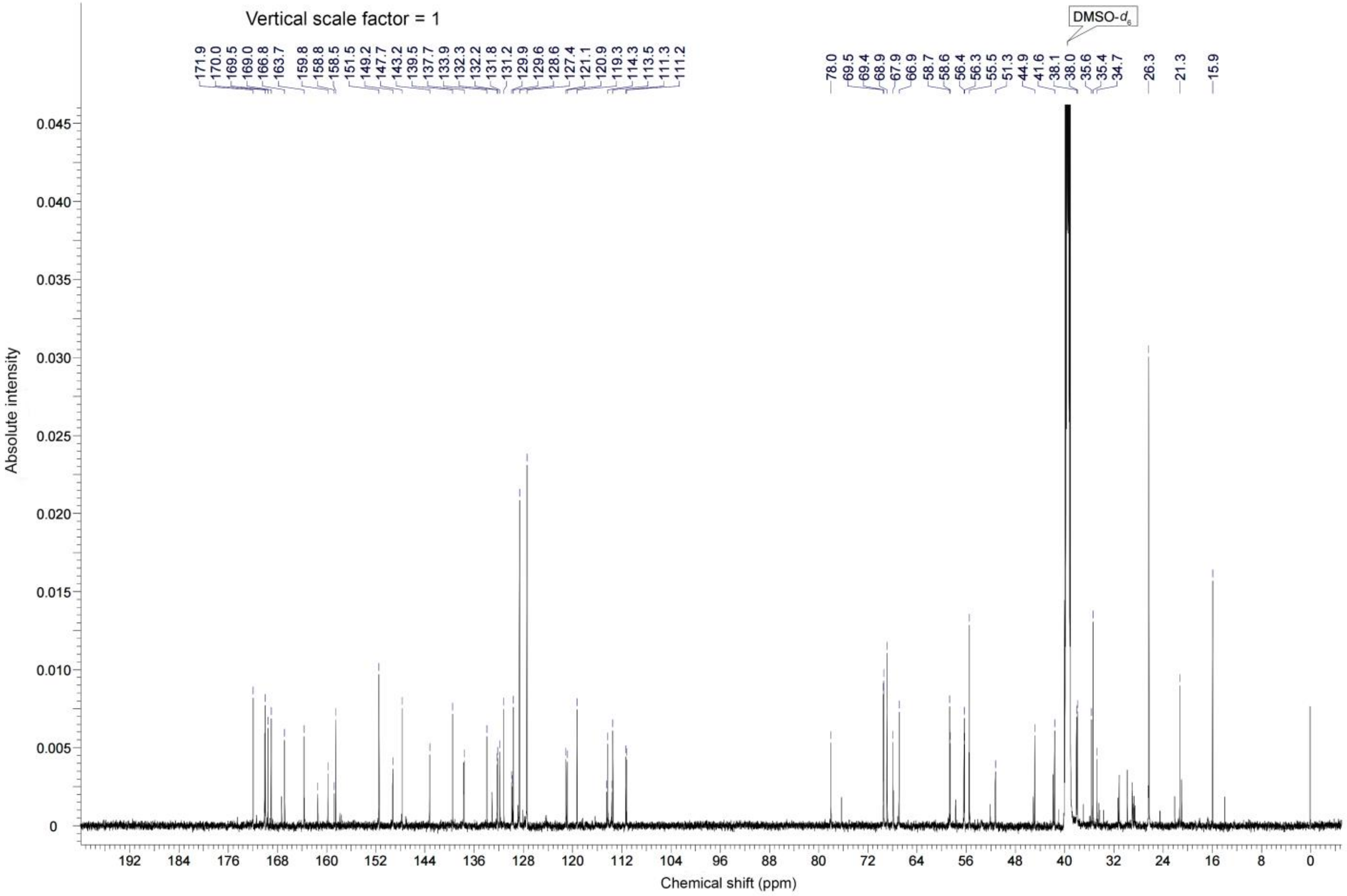
Vertical scale factor = 1
DMSO-d_6
171.9
170.0
169.5
169.0
166.8
163.7
159.8
158.8
158.5
151.5
149.2
147.7
143.2
139.5
137.7
133.9
132.3
132.2
131.8
131.2
129.9
129.6
128.6
127.4
121.1
120.9
119.3
114.3
113.5
111.3
111.2
78.0
69.5
69.4
68.9
67.9
66.9
58.7
58.6
56.4
56.3
55.5
51.3
44.9
41.6
38.1
38.0
35.6
35.4
34.7
26.3
21.3
15.9
Absolute intensity
Chemical shift (ppm)
